# Wnt5a‐Induced Exosomes From Bone Marrow Mesenchymal Stem Cells Promote Spinal Cord Injury Repair by Modulating Immune Cell Phenotypes and Alleviating Neuroinflammation via the NF‐κB Pathway

**DOI:** 10.1002/cns.70834

**Published:** 2026-03-26

**Authors:** Dan Luo, Enhui Feng, Ling Chen, Ke Ni, Sheng Luo, Lin Ma, Yuting Wu, Zhifeng Hu, Dingkun Lin, Binshan Zhang, Chaoyi Yin, Da Guo, Chaolun Liang, Xing Li

**Affiliations:** ^1^ State Key Laboratory of Traditional Chinese Medicine Syndrome, State Key Laboratory of Dampness Syndrome of Chinese Medicine, Department of Orthopedic Surgery The Second Affiliated Hospital of Guangzhou University of Chinese Medicine Guangzhou Guangdong China; ^2^ Spinal Minimally Invasive Department, Spinal Degenerative Disease Prevention and Treatment Research Team The Second Affiliated Hospital of Guangzhou University of Chinese Medicine Guangzhou Guangdong China; ^3^ Laboratory of Osteology and Traumatology of Traditional Chinese Medicine, Lingnan Medical Research Center Guangzhou University of Chinese Medicine Guangzhou Guangdong China; ^4^ Department of Orthopedics The Second Affiliated Hospital of Guangzhou University of Chinese Medicine Guangzhou Guangdong China; ^5^ Guangzhou University of Chinese Medicine Guangzhou Guangdong China; ^6^ The Second Clinical College of Guangzhou University of Chinese Medicine Guangzhou Guangdong China; ^7^ Dalian Ocean University Dalian Liaoning China; ^8^ College of Physical Education and Health Guangzhou University of Chinese Medicine Guangzhou Guangdong China; ^9^ Zhongshan Hospital of Traditional Chinese Medicine Affiliated to Guangzhou University of Chinese Medicine Zhongshan Guangdong China; ^10^ Department of Orthopedics Dongguan Hospital of Guangzhou University of Chinese Medicine Dongguan Guangdong China; ^11^ Department of Orthopedics (Articular Surgery) The Zhuhai Hospital of Guangdong Province Hospital of Chinese Medicine Zhuhai Guangdong China

**Keywords:** exosome, inflammation, neuronal differentiation, NF‐κB, spinal cord injury, stem cell, Wnt5a

## Abstract

**Objective:**

Spinal cord injury is a devastating neurological disorder. The transplantation of mesenchymal stem cell‐derived exosomes has shown great promise. Wnt5a has been reported to promote neuronal differentiation and spinal cord regeneration. However, its mechanistic role when delivered via exosomes remains unclear. In the present study, the function and underlying mechanism of Wnt5a in promoting neuronal differentiation and spinal cord repair were investigated using bone marrow mesenchymal stem cell‐derived exosomes.

**Methods:**

This study initially constructed Wnt5a‐overexpressing BMSCs and isolated and characterized their exosomes. Subsequently, in vitro experiments were conducted to detect markers of neurons, astrocytes, and microglia. This was followed by high‐throughput sequencing to analyze related pathways. Finally, in vivo experiments were performed, in which rats were divided into the Sham group, the SCI group, the Exo group, and the pLV‐Exo‐Wnt5a group. The effects of exosomes in vivo were analyzed through histological, behavioral, electrophysiological, western blot, PCR, and immunofluorescence assays.

**Results:**

Our results demonstrated that Wnt5a‐enriched BMSC‐Exos significantly enhanced the proliferation and neuronal differentiation of neural stem cells, while suppressing astrocyte formation. High‐throughput RNA sequencing revealed an association between Wnt5a and the NF‐κB signaling pathway. Intervention with lipopolysaccharide confirmed that Wnt5a exerts a suppressive effect on this pathway. In vivo, the transplantation of Wnt5a‐modified BMSC‐Exos facilitated the polarization of microglia towards an anti‐inflammatory M2 phenotype, promoted neurogenesis, reduced astrocyte accumulation, improved spinal cord tissue architecture, and led to better motor function recovery.

**Conclusion:**

Collectively, these findings indicate that Wnt5a enhances the neuroregenerative potential of BMSC‐Exos by modulating immune responses and suppressing neuroinflammation, likely through inhibiting the NF‐κB signaling pathway.

## Graphical abstract

Wnt5a positively influences neuronal regeneration after SCI via BMSC‐Exos, potentially through immune regulation and suppression of neuroinflammation. Transplantation of Wnt5a‐modified BMSC‐Exos could enhance tissue repair and the recovery of motor function after SCI in vivo, highlighting their potential in treating SCI.

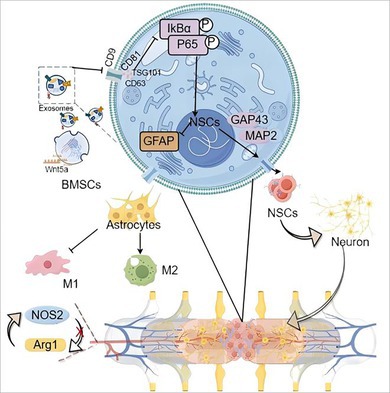

**Abbreviations**
BBB: Basso‐Beattie‐Bresnahan
BCA: bicinchoninic acid
BMSC: bone marrow mesenchymal stem cell
DEGs: differentially expressed genes
EGF: epidermal growth factor
FBS: fetal bovine serum
FGF: fibroblast growth factor
HE: hematoxylin and eosin
HRP: horseradish peroxidase
LPS: lipopolysaccharide
MEP: motor evoked potentials
MSC‐Exo: mesenchymal stem cell‐derived exosome
NC: negative control
NF‐κB: nuclear factor‐κB
NSC: neural stem cell
PBS: phosphate‐buffered saline
PCA: principal component analysis
PFA: paraformaldehyde
PVDF: polyvinylidene difluoride
RIPA: ristocetin‐induced platelet aggregation
RT‐qPCR: reverse transcription quantitative polymerase chain reaction
SCI: spinal cord injury
SD: Sprague Dawley
SDS‐PAGE: sodium dodecyl sulfate polyacrylamide gel electrophoresis
SEM: standard error of measurement
WB: western blotting

## 1 Introduction

Spinal cord injury (SCI) is a significant neurological condition. It frequently leads to sensory and motor dysfunction of the limbs, loss of reflexes, disturbances in bowel and bladder control, paralysis, and in severe cases, death [1, 2]. SCI imposes considerable physical, psychological, and economic burdens on patients and their families [3].

After the occurrence of SCI, the spinal cord environment undergoes destruction, resulting in the loss of a large number of cells. The limited role of resident endogenous neural stem cells in SCI repair has led to the consideration of exogenous cell transplantation as a potential solution. The transplantation of mesenchymal stem cells (MSCs) has been established as an effective therapeutic strategy for SCI [4]. MSCs not only possess self‐renewal and differentiation capabilities, effectively replenishing lost cells, but also promote angiogenesis, neurogenesis, matrix remodeling, microenvironment improvement, and neuroprotection [5, 6]. However, clinical applications are limited by challenges such as high post‐transplantation apoptosis rates, unstable differentiation potential, and risks of tumorigenicity and teratogenicity [7].

The therapeutic effects of MSCs were often attributed to their engraftment and differentiation capabilities in the past. However, a study has found that the engraftment time of MSCs is typically too short to exert a significant impact [8]. Other research has indicated that < 1% of MSCs survive for more than 1 week after systemic administration [9]. Increasing evidence suggests that the reparative and regenerative effects of MSCs are primarily mediated through paracrine mechanisms involving exosomes (Exos) [10]. Exos are extracellular nanovesicles, typically 40–100 nm in diameter, spherical in shape, and enclosed by a lipid bilayer membrane [11, 12]. They carry bioactive molecules and play a crucial role in intercellular communication by transferring biological information and cargo. Recent studies have shown that MSC‐derived exosomes (MSC‐Exos) can mimic the functional properties of their parent cells, with low immunogenicity and the potential for administration via multiple delivery routes [13]. These features make Exos a promising alternative to stem cell‐based therapies, potentially redefining the next frontier in regenerative medicine. However, strategies to enhance the therapeutic efficacy of Exos remain under intensive investigation.

The Wnt protein family is intricately associated with the differentiation, proliferation, and migration of neuronal cells [14, 15]. Previous work from our group demonstrated that Wnt5a promotes neuronal differentiation while reducing astrocyte formation, ultimately improving spinal cord tissue integrity and motor function in rat models [16]. However, it remains unclear whether such effects can be achieved through exosomal delivery. Furthermore, MSCs have been proven to facilitate tissue repair by inhibiting nuclear factor‐κB (NF‐κB) activation and attenuating the release of inflammatory cytokines [17, 18]. MSC‐Exos can inhibit inflammation by altering the phenotype of microglia or macrophages [19]. Nakao et al. [20] reported that Wnt5a regulates the expression of receptor activator of NF‐κB ligand. In previous studies, numerous investigations have confirmed that Wnt5a exerts a positive effect on the differentiation and biological functions of various stem cells [21, 22, 23, 24]; further evidence has demonstrated that Wnt5a can promote the directed differentiation of neural stem cells into neurons [25, 26]. Despite these findings, it remains unclear whether the neuroregenerative role of Wnt5a, particularly its ability to enhance neuronal differentiation and limit astrocyte, is mediated via immune modulation and transmitted through Exos. Therefore, this study was aimed to assess whether MSC‐Exos exerted effects consistent with their donor cells through in vitro experiments, and explore the underlying mechanisms using transcriptomic analysis, and finally validate the findings in vivo, thereby providing mechanistic insights and experimental evidence to support the development of more effective Exo‐based regenerative therapies for SCI.

## 2 Methods

### 2.1 Cells and Cultures

Two cell types were used in this study. Bone marrow mesenchymal stem cells (BMSCs), known for their robust differentiation potential and ability to promote motor function recovery, were chosen as the source for Exo secretion [27]. Neural stem cells (NSCs) and microglia were used for in vitro experiments. BMSCs were derived from the bone marrow of Sprague–Dawley (SD) rats and have been characterized in previous research [16], while NSCs and microglia were isolated from the spinal cords of neonatal rats and characterized in this study. BMSCs were cultured in Dulbecco's Modified Eagle Medium (DMEM; Thermo Fisher) supplemented with 10% fetal bovine serum (FBS; Thermo Fisher) and 100 U/mL penicillin–streptomycin (Thermo Fisher), maintained at 37°C in a 5% CO_2_ incubator. The culture medium was refreshed every 72 h [26]. NSCs and microglia were maintained in a proliferation medium consisting of DMEM/F12 (Thermo Fisher), 2% B‐27 supplement (Thermo Fisher), 2% N‐2 supplement (Thermo Fisher), 10 ng/mL epidermal growth factor (EGF; PeproTech), 10 ng/mL basic FGF (bFGF; PeproTech), and 1% penicillin–streptomycin (100 U/mL). Cultures were incubated at 37°C with 5% CO_2_, and the medium was partially replaced every other day [28]. NSCs were identified by immunofluorescence staining.

### 2.2 Generation of Wnt5a‐Overexpressing BMSCs via Lentiviral Vectors

A lentiviral vector encoding rat Wnt5a was constructed using rat genomic DNA as a template, with an empty lentiviral vector used as a negative control (NC). The constructed vectors were co‐transfected into 293T cells along with packaging plasmids. After 8 h of incubation, the medium was replaced with complete culture medium and incubated for an additional 48 h. The supernatant was then collected, filtered, concentrated, and resuspended to achieve the appropriate multiplicity of infection (MOI). BMSCs were transduced with the lentivirus and divided into three groups: the control (Con), NC, and mimic groups. The NC group received the empty lentiviral vector, while the mimic group was transduced with the Wnt5a‐overexpressing vector. The expression of Wnt5a in BMSCs was confirmed by Western blotting (WB) and reverse transcription quantitative polymerase chain reaction (RT‐qPCR).

### 2.3 Isolation and Identification of BMSC‐Derived Exos (BMSC‐Exos)

Exos were isolated from the culture supernatants of two groups of BMSCs (NC group and the mimic group), and labeled as the Exo group and the pLV‐Exo‐Wnt5a group, respectively. Unmodified BMSCs served as the Con group. Fourth‐passage BMSCs at approximately 80% confluence were cultured in serum‐free medium for 48 h prior to Exo isolation. Exos were isolated using a standard ultracentrifugation protocol. Briefly, the supernatant was first centrifuged at 300 × *g* for 10 min, followed by 2000 × *g* for another 10 min to remove cells and apoptotic bodies. The supernatant was then filtered through a 0.22 μm membrane and centrifuged at 10,000 × *g* for 30 min to eliminate cellular debris. The clarified supernatant was ultracentrifuged at 100,000 × *g* for 70 min. The resulting pellet was washed with phosphate‐buffered saline (PBS) and centrifuged again at 100,000 × *g* for 70 min. The final Exo pellet was resuspended in PBS. All centrifugation steps were performed at 4°C. Isolated Exos were either stored at −80°C or used immediately for subsequent experiments.

Exo morphology was examined using transmission electron microscopy, and particle size distribution was analyzed using a nanoparticle tracking analysis. WB analysis was performed to assess the expression of exosome markers (CD63, CD81, CD9, TSG101) and endoplasmic reticulum marker (calnexin) in both the Exo and pLV‐Exo‐Wnt5a groups, using BMSCs as a control.

### 2.4 In Vitro Experiments

Well‐growing second‐generation NSCs were seeded in 24‐well plates and cultured in differentiation medium composed of DMEM/F12, 2% B‐27, 2% N‐2, 10 ng/mL FGF, 10 ng/mL bFGF, and 2% FBS. Cultures were maintained at 37°C in a 5% CO_2_ incubator. NSCs were divided into three groups: the Con group (cultured in differentiation medium), the Exo group (supplemented with BMSC‐Exos), and the pLV‐Exo‐Wnt5a group (supplemented with Wnt5a‐overexpressing BMSC‐Exos). Each group consisted of eight replicates. Following 7 days of continuous differentiation, neuronal and astrocytic markers were assessed by immunofluorescence staining and WB to evaluate cellular differentiation across the three groups.

An inflammatory model was established by inducing microglial cells with lipopolysaccharide (LPS; 1 μg/mL; Sigma Aldrich) [29]. Briefly, the microglial cells were divided into four groups: the Con group (cultured with medium), the LPS group (treated with LPS), the Exo group (treated with LPS + BMSC‐Exos), and the pLV‐Exo‐Wnt5a group (treated with LPS + Wnt5a‐overexpressing BMSC‐Exos).

### 2.5 High‐Throughput RNA Sequencing (RNA‐Seq) and Bioinformatics Analysis

Total RNA was extracted using TRIzol reagent following the manufacturer's instructions, and its purity, concentration, and integrity were assessed. Complementary DNA (cDNA) libraries were constructed using the VAHTS Universal V6 RNA‐seq Library Prep Kit, following the manufacturer's guidelines. Sequencing was performed on the Illumina NovaSeq 6000 platform, generating 150 bp paired‐end reads. After removing low‐quality sequences, bioinformatics analysis was conducted. HISAT2 was used for sequence alignment and fragments per kilobase of transcript per million mapped reads (FPKM) calculations. Principal component analysis (PCA) was conducted with R version 3.2.0 to evaluate sample reproducibility, and differential gene expression analysis was carried out using DESeq2. Differentially expressed genes (DEGs) were identified based on a *Q*‐value < 0.05 and a fold change (FC) > 2 or < 0.5. Hierarchical clustering and Kyoto Encyclopedia of Genes and Genomes (KEGG) pathway enrichment analyses of DEGs were conducted using R software.

### 2.6 Animals

All animal procedures were approved by the Experimental Animal Management Ethics Committee of Guangzhou University of Chinese Medicine (Approval number: 2023013). This study exclusively used male specific‐pathogen‐free Sprague Dawley (SD) rats, weighing 180–220 g, sourced from the Experimental Animal Center of Guangzhou University of Chinese Medicine. The sample size for this experiment was determined based on our previous research [26]. The rats were housed under standard laboratory conditions: 4 animals per cage, temperature maintained at 20°C–25°C, 12‐h light/dark cycle, and relative humidity of 50%–65%. All animals were acclimated for 1 week prior to the start of the experiments. Dead rats would be excluded.

### 2.7 Establishment of the Rat Model of SCI

A rat model of SCI was established using Allen's method [30]. Rats were anesthetized with 3% pentobarbital sodium and placed in a fixed position. Hair around the T10 spinal segment was removed, and a 4 cm longitudinal incision was made to expose the T9–T11 vertebrae. The spinous processes and laminae were removed to expose the T10 segment of the spinal cord. A calibrated impactor was used to strike the spinal cord at T10 (impact speed: 1.2 m/s; depth: 1.0 mm; dwell time: 85 ms). Successful modeling was indicated by local hyperemia and transient convulsions or trembling of the trunk, hind limbs, or tail. Rats in the sham group underwent only spinal cord exposure.

### 2.8 In Vivo Experiments

A total of 60 rats were randomly assigned to four groups (*n* = 15 per group): Sham group, SCI group, Exo group, and pLV‐Exo‐Wnt5a group using a computer‐generated randomization sequence (GraphPad Prism 9.0) to minimize bias (the latter three groups were all successfully modeled rats). All treatments were administered via tail vein injection. One day post‐surgery, rats in the Sham and SCI groups were injected with 200 μL of PBS, while those in the Exo and pLV‐Exo‐Wnt5a groups received an equal volume of PBS containing Exos. Specifically, the Exo group received BMSC‐Exos, and the pLV‐Exo‐Wnt5a group received Exos from Wnt5a‐overexpressing BMSCs.

### 2.9 Acquisition and Preservation of SCI

On day 21 post‐intervention, rats were euthanized and segments of the injured spinal cord were collected. A segment of the damaged spinal cord tissue was cryopreserved in liquid nitrogen (−196°C) for future WB analysis. The remaining tissue was dehydrated and embedded in paraffin, then sectioned at a thickness of 5 μm for histopathological staining.

### 2.10 Histopathological Staining and Observation

Formaldehyde‐fixed spinal cord tissues were subjected to graded dehydration, embedded in paraffin, and sectioned. One portion of sections was stained with hematoxylin and eosin (HE), while another portion underwent cresyl violet staining. After clearing with xylene and mounting with neutral balsam, tissue morphology and Nissl body distribution were examined and imaged using a light microscope. Histological evaluations were independently performed by two researchers and then summarized.

### 2.11 Animal Behavioral Assessment

The Basso‐Beattie‐Bresnahan (BBB) locomotor scale and footprint test were used to evaluate hindlimb locomotor function in SCI rats [31, 32]. Assessments were conducted on days 1, 3, 7, 14, and 21 post‐intervention. For each evaluation, rats were placed in an open field and allowed to move freely for 15 min, after which hindlimb locomotor ability was scored using the BBB scale, which ranged from 0 (complete paralysis) to 21 (normal function). On day 21, a footprint test was conducted by coating the rats' hind paws with dye and allowing them to walk along a 7.5 cm × 100 cm runway lined with white paper. Gait patterns and limb coordination were recorded and analyzed from the resulting footprints.

### 2.12 Electrophysiological Testing

To objectively evaluate functional recovery following SCI, motor evoked potentials (MEPs) were recorded in SCI rats on day 21 using an electrophysiological monitoring system [33]. Under anesthesia, the stimulating electrode was placed subcutaneously along the midline of the rat's skull. The recording electrode was positioned within the tendon of the biceps femoris muscle, the reference electrode was inserted into the distal tendon of the hindlimb muscle, and the ground electrode was placed subcutaneously on the dorsal skin. A single square‐wave stimulus (5 ms, 1 mV) was delivered, and the waveform and amplitude of the MEPs were recorded. All behavioral assessments and electrophysiological tests were performed by two independent researchers.

### 2.13 Western Blotting (WB) Analysis

Following tissue, cell, and Exo processing, total protein was extracted using radioimmunoprecipitation assay (RIPA) buffer containing phosphatase and protease inhibitors (1 μg/mL; Sigma‐Aldrich). The protein concentrations were then quantified using a bicinchoninic acid (BCA) protein assay kit. Equal amounts of protein (20 μg per sample) were separated by 10% sodium dodecyl sulfate polyacrylamide gel electrophoresis (SDS‐PAGE) and transferred onto polyvinylidene difluoride (PVDF) membranes. Membranes were blocked with NcmBlot fast blocking buffer and probed with the following primary antibodies at 4°C: CD9 (1:1000; Abcam), CD81 (1:1000; Abcam), CD63 (1:1000; Abcam), TSG101 (1:1000; Abcam), calnexin (1:1000; Abcam), Ki67 (1:1000; Cell Signaling Technology), NeuN (1:500; Boster Bioengineering), GFAP (1:500; Boster Bioengineering), P65 (1:1000; Abcam), phosphorylated P65 (p‐P65) (1:1000; Abcam), IκBα (1:1000; Abcam), p‐IκBα (1:1000; Abcam), Arg1 (1:1000; Cell Signaling Technology), NOS2 (1:1000; Cell Signaling Technology), MAP2 (1:500; Boster Bioengineering), GAP43 (1:1000; NOVUS), and GAPDH (1:1000; Cell Signaling Technology). GAPDH served as the internal control, except for p‐P65 and p‐IκBα, which were normalized to their total protein counterparts (P65 and IκBα, respectively). After overnight incubation, membranes were washed with Tris‐buffered saline with Tween (TBST) and incubated with horseradish peroxidase (HRP)‐conjugated secondary antibodies (1:1000) for 120 min at room temperature. Protein bands were visualized using the ChemiDoc MP imaging system (Bio‐Rad), and band intensities were determined using ImageJ software. All steps involving phosphorylated proteins were performed at 4°C to preserve protein integrity.

### 2.14 RT‐qPCR Analysis

Following cell processing, total RNA was extracted using TRIzol reagent (Thermo Fisher). RT‐PCR was performed using the First Strand cDNA Synthesis Kit and the PrimeScript RT Kit (Thermo Fisher) using a SYBR Green‐based system (Toyobo, Japan Ltd., Japan) under the following cycling conditions: initial denaturation at 95°C for 2 min; 40 cycles of denaturation at 95°C for 5 s, annealing at 60°C for 30 s, and extension at 72°C for 45 s. Relative mRNA expression levels were calculated using the 2^−ΔΔ*Ct*^ method. Primer sequences used in this study are provided in Table 1.

**TABLE 1:** Primers used for target amplification in this study.

Gene	Primer sequence (5′‐3′)
Wnt5a	Forward: CGTGGCTATGACCAGTTTAAG Reverse: CCACAATCTCCGTGCACTT
GFAP	Forward: GCAGAGGACCACAGCCCTCCAGA Reverse: TCCAGGTAGGTTCATGG
NeuN	Forward: TCATCGTCAAATCTGAGACTCTGGAG Reverse: CGGCGCAGCTCTCTCGTCCT
IL‐10	Forward: GCCCTTTGCTATGGTGTC Reverse: TCTCCCTGGTTTCTCTTCC
IL‐6	Forward: AACCGCTATGAAGTTCCTCTCTG Reverse: AGTGCCTTTGATTCCACATTG
Arg1	Forward: CTCCAAGCCAAAGTCCTTAGAG Reverse: AGGAGCTGTCATTAGGGACATC
TGF‐β	Forward: TGGGGACTTCTTGGCACT Reverse: ATAGGGGCGTCTGAGGAAC

### 2.15 Immunofluorescence Staining and Analysis

Cells were fixed in 4% paraformaldehyde (PFA) for 2 h. Tissue sections were dewaxed with xylene and rehydrated through graded ethanol. Antigen retrieval was performed using 10 mM sodium citrate buffer (pH 6.0), followed by permeabilization with 0.3% Triton X‐100 for 60 min at room temperature. Subsequently, sections were blocked with 10% goat serum for 1 h and incubated overnight at 4°C with the following primary antibodies: Nestin (1:200; Abcam), β‐III‐tubulin (1:200; Abcam), BrdU (1:500; Sigma‐Aldrich), Ki67 (1:500; Cell Signaling Technology), NeuN (1:200; Boster Bioengineering), GFAP (1:600; Boster Bioengineering), Iba1 (1:100; Abcam), Arg1 (1:200; Cell Signaling Technology), NOS2 (1:200; Cell Signaling Technology), MAP2 (1:200; Boster Bioengineering), and GAP43 (1:200; NOVUS). The following day, sections were incubated with Alexa Fluor‐conjugated secondary antibodies (1:300; Invitrogen), diluted in PBS, according to the corresponding primary antibodies. Cell nuclei were counterstained with 4′,6‐diamidino‐2‐phenylindole (DAPI). Fluorescence signals were observed and imaged using a fluorescence microscope.

### 2.16 Statistical Analysis

Data analyses were conducted through SPSS version 16.0 (SPSS Inc., Chicago, IL, USA). All data were expressed with mean ± standard error of the mean (SEM). For comparisons between two groups, Student's *t*‐test was carried out; one‐way analysis of variance (ANOVA) was applied for multiple group comparisons. Statistical significance was determined using a threshold of *p* < 0.05.

## 3 Results

### 3.1 Effective Overexpression of Wnt5a by BMSCs

**FIGURE 1:**
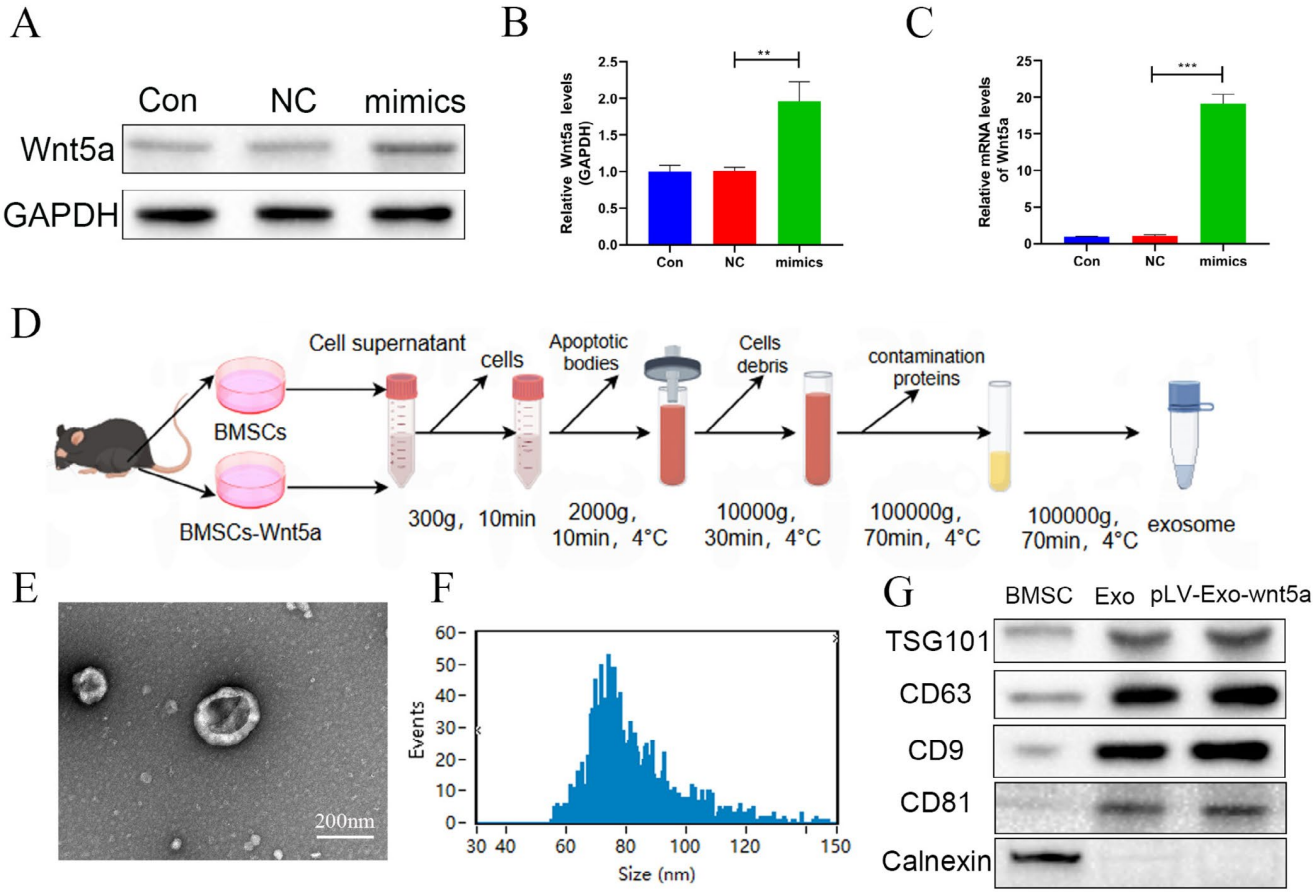
Extraction and identification of Wnt5a‐modified BMSC‐Exos. (A–C) BMSCs can efficiently overexpress Wnt5a. (D) The extraction process of Exos. (E) The morphology of Exos. (F) The particle size distribution of Exos. (G) Identification of Exos. Data are presented as mean ± SEM, **p < 0.01, ***p < 0.001 compared to the adjacent group.

To verify the overexpression of Wnt5a in BMSCs, this study first evaluated Wnt5a expression levels across three BMSC groups subjected to different interventions. The results demonstrated that BMSCs effectively overexpressed Wnt5a, independent of the cells' endogenous regulation (Figure 1A–C).

### 3.2 Identification and Characterization of BMSC‐Exos

The extraction process of BMSC‐Exos is illustrated in Figure 1D. The isolated BMSC‐Exos appeared concave cup‐shaped in morphology (Figure 1E), with particle sizes ranging between 50 and 150 nm (Figure 1F). WB analysis demonstrated significantly higher expression levels of the exosomal markers (CD63, CD81, CD9, TSG101) in the samples compared to those in BMSC samples, while the expression of calnexin was nearly undetectable. These expression levels were not influenced by Wnt5a overexpression (Figure 1G).

### 3.3 Wnt5a‐Modified BMSC‐Exos Enhanced the Proliferation and Neuronal Differentiation of NSCs

**FIGURE 2:**
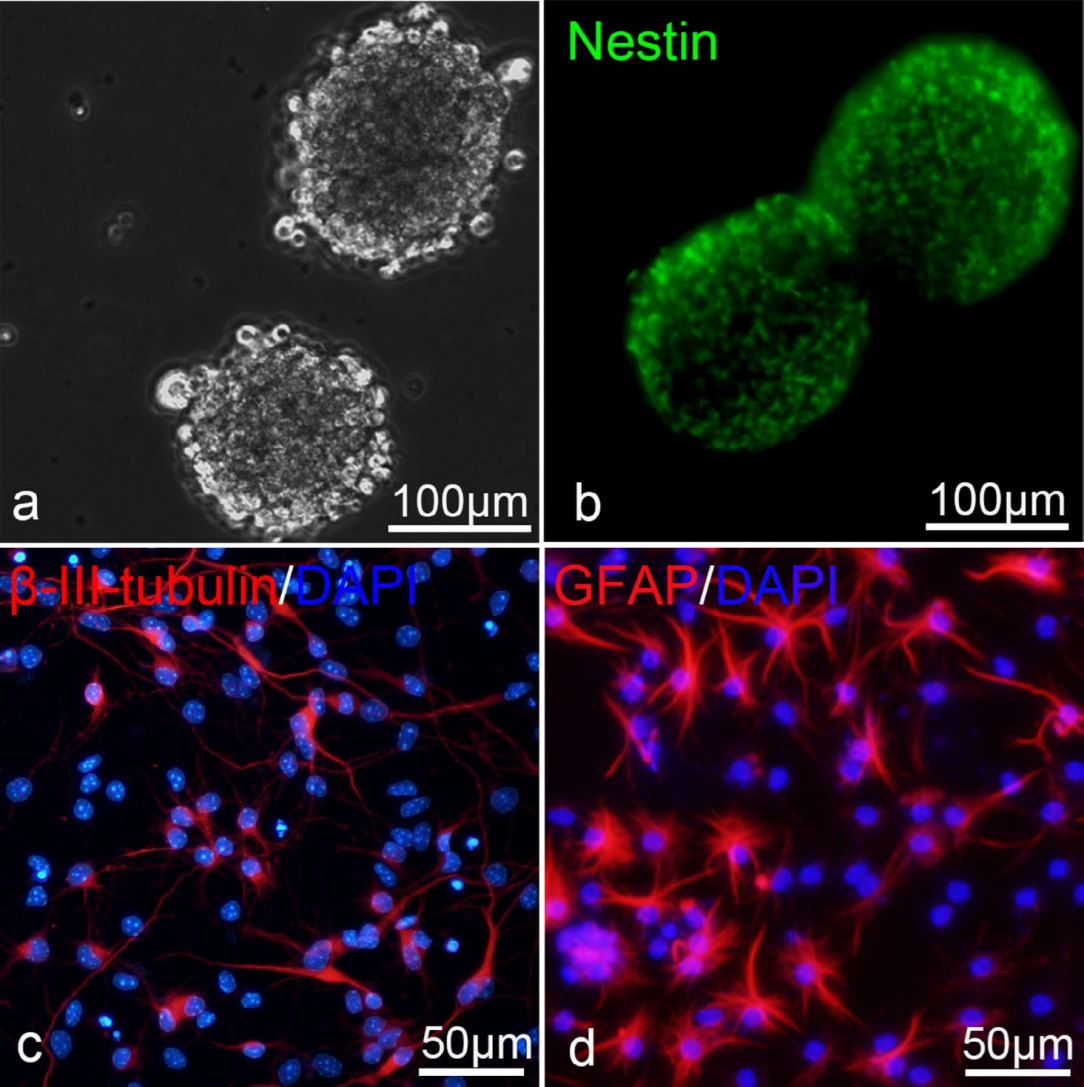
Identification of NSCs using immunofluorescence and confirmation of their ability to differentiate into neurons. (a) NSCs under microscopy. (b–d) NSCs were confirmed and their differentiation into neurons was demonstrated by immunofluorescence.

**FIGURE 3:**
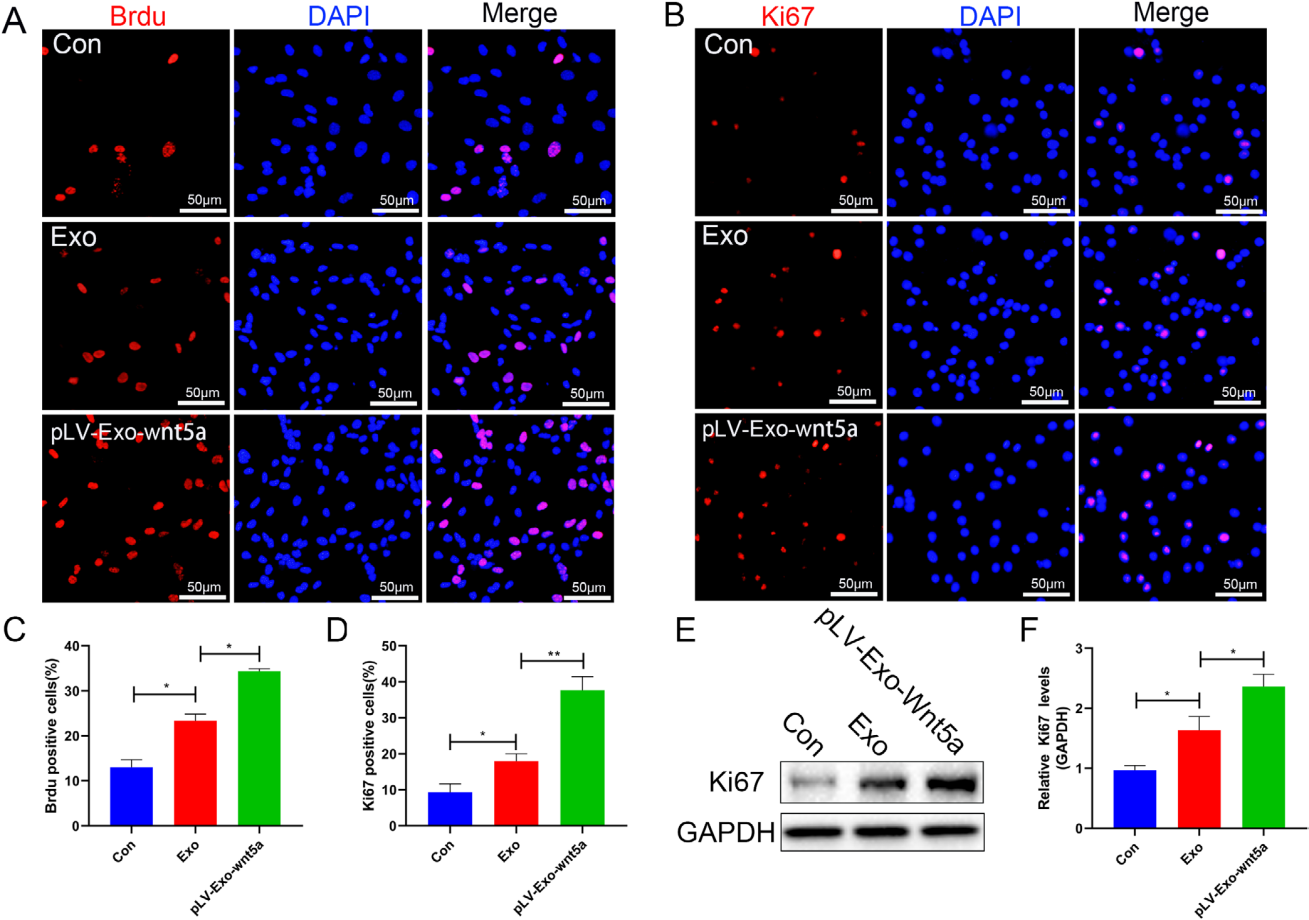
Wnt5a‐modified BMSC‐Exos promote the proliferation of NSCs. (A–D) Cell proliferation markers BrdU and Ki67 in each group were determined by immunofluorescence. (E, F) The expression of Ki67 protein in the cells was detected by Western blot. Data are presented as mean ± SEM, *p < 0.05, **p < 0.01 compared to the adjacent group.

**FIGURE 4:**
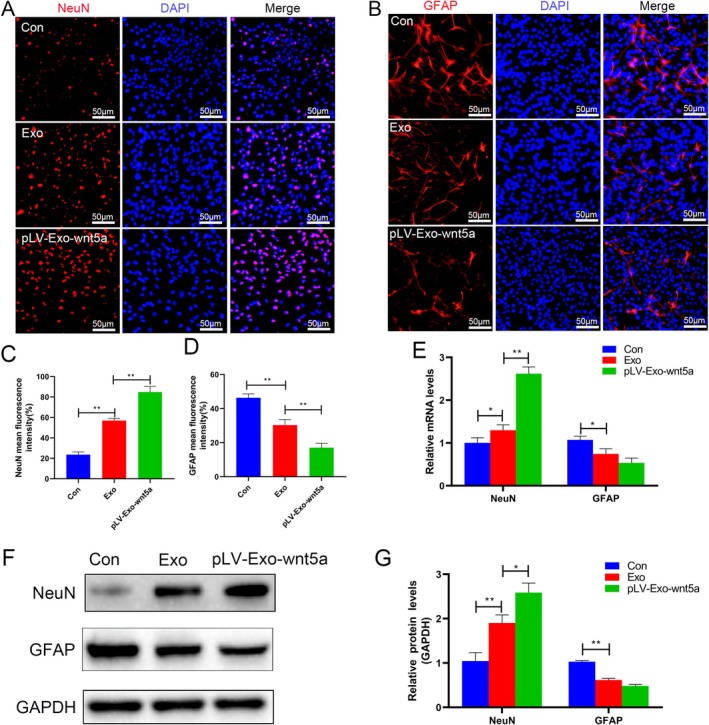
Wnt5a‐modified BMSC‐Exos promote the differentiation of NSCs into neurons and reduce the production of astrocytes. (A–D) The neuronal marker NeuN and the astrocyte marker GFAP were determined by immunofluorescence assay. (E) The mRNA levels of NeuN and GFAP in each group of cells were detected by RT‐qPCR. (F, G) The expression of NeuN and GFAP proteins in the cells was examined by Western blot. Data are presented as mean ± SEM, *p < 0.05, **p < 0.01 compared to the adjacent group.

NSCs were first identified and confirmed to possess the potential to differentiate into mature neurons (Figure 2). In this study, treatment with BMSC‐Exos significantly enhanced the fluorescence intensity of proliferation markers BrdU and Ki67, as well as the protein expression level of Ki67 in NSCs compared to BMSCs (*p* < 0.05). Wnt5a‐modified Exos further amplified these effects (*p* < 0.05) (Figure 3), suggesting that BMSC‐Exos promote cell proliferation and that Wnt5a overexpression further enhances this effect. To assess differentiation, the expression of the neuronal marker NeuN and the astrocyte marker GFAP was evaluated following directed culture of NSCs. Exo transplantation increased the fluorescence intensity of NeuN and decreased the fluorescence intensity of GFAP with statistical significance (both *p* < 0.05). The changes observed in the pLV‐Exo‐Wnt5a group were more significant than those in the Exo group (*p* < 0.05). Additionally, the mRNA levels and protein expression levels of NeuN and GFAP were consistent with the results of the immunofluorescence assay (*p* < 0.05) (Figure 4). Collectively, these findings indicate that BMSC‐Exos promote the proliferation and neuronal differentiation of NSCs while suppressing astrocyte formation, and that Wnt5a overexpression enhances these effects.

### 3.4 Transcriptomic Sequencing Analysis of BMSC‐Exos

**FIGURE 5:**
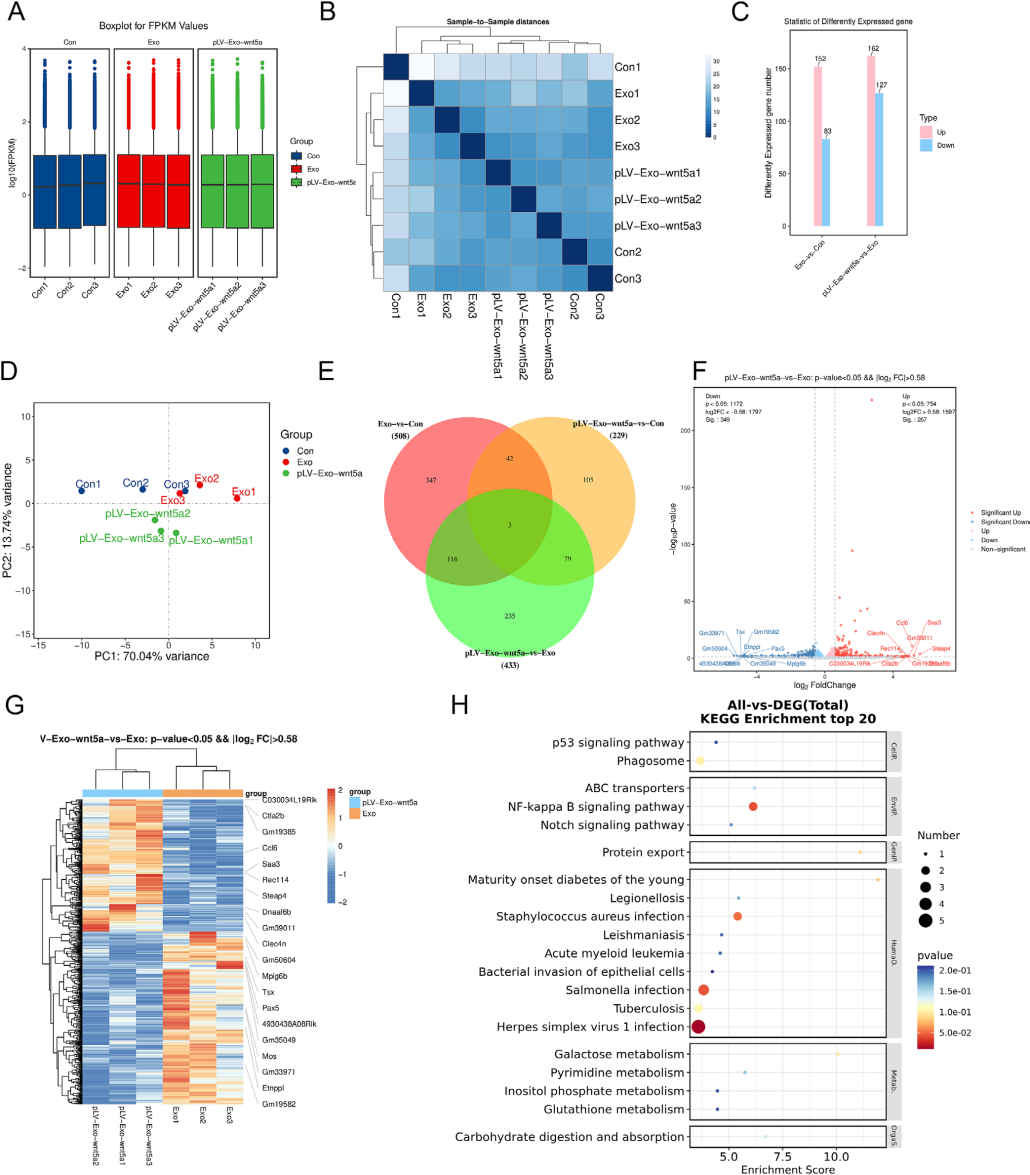
Transcriptomic sequencing results for samples from each group. (A) Gene expression distribution of samples from the Con, Exo, and pLV‐Exo‐Wnt5a groups is relatively consistent. (B) The expression values within each group show high similarity, while similarity between groups is lower. (C) Number of differential metabolites among the Con, Exo, and pLV‐Exo‐Wnt5a groups. “Up” refers to the number of significantly upregulated genes, while “down” refers to the number of downregulated genes. (D) Principal Component Analysis reveals significant differences among samples from different groups. (E) A Venn diagram illustrating the common differentially expressed genes among the Con, Exo, and pLV‐Exo‐Wnt5a groups. (F) A volcano plot of differentially expressed genes between the Exo and pLV‐Exo‐Wnt5a groups, with blue dots representing downregulated genes and red dots representing upregulated genes. (G) A clustered heatmap of differentially expressed genes in the Con, Exo, and pLV‐Exo‐Wnt5a groups. The color scale indicates relative abundance, with blue representing significant downregulation and red representing significant upregulation. (H) KEGG enrichment analysis.

High‐throughput RNA‐seq was conducted on samples collected from the Con, Exo, and pLV‐Exo‐Wnt5a groups. The normalization of expression values was achieved using FPKM for subsequent analysis (Figure 5A). The results of similarity analysis together with PCA showed high intra‐group consistency and low inter‐group similarity, supporting the reliability of the sequencing data (Figure 5B). Differential gene expression analysis identified 152 upregulated and 83 downregulated genes in the Exo group compared to the Con group. When comparing the pLV‐Exo‐Wnt5a group to the Exo group, 162 genes were upregulated and 127 were downregulated (Figure 5C). Three DEGs were consistently observed across the three groups (Figure 5E). Using a threshold of absolute log_2_FC > 0.58 and *p* < 0.05, 616 DEGs were identified between the pLV‐Exo‐Wnt5a and Exo groups, including 267 upregulated and 349 downregulated genes (Figure 5F,G). KEGG enrichment analysis revealed that these DEGs were predominantly involved in biological activities like autophagy, immunological response, and receptor–ligand interactions. NF‐κB signaling pathway, which is closely associated with inflammatory responses, cell proliferation, and differentiation, was among the significantly enriched pathways (Figure 5H).

### 3.5 Wnt5a‐Modified BMSC‐Exos Regulated Neurons and Astrocytes via Inhibition of NF‐κB Signaling Pathway

**FIGURE 6:**
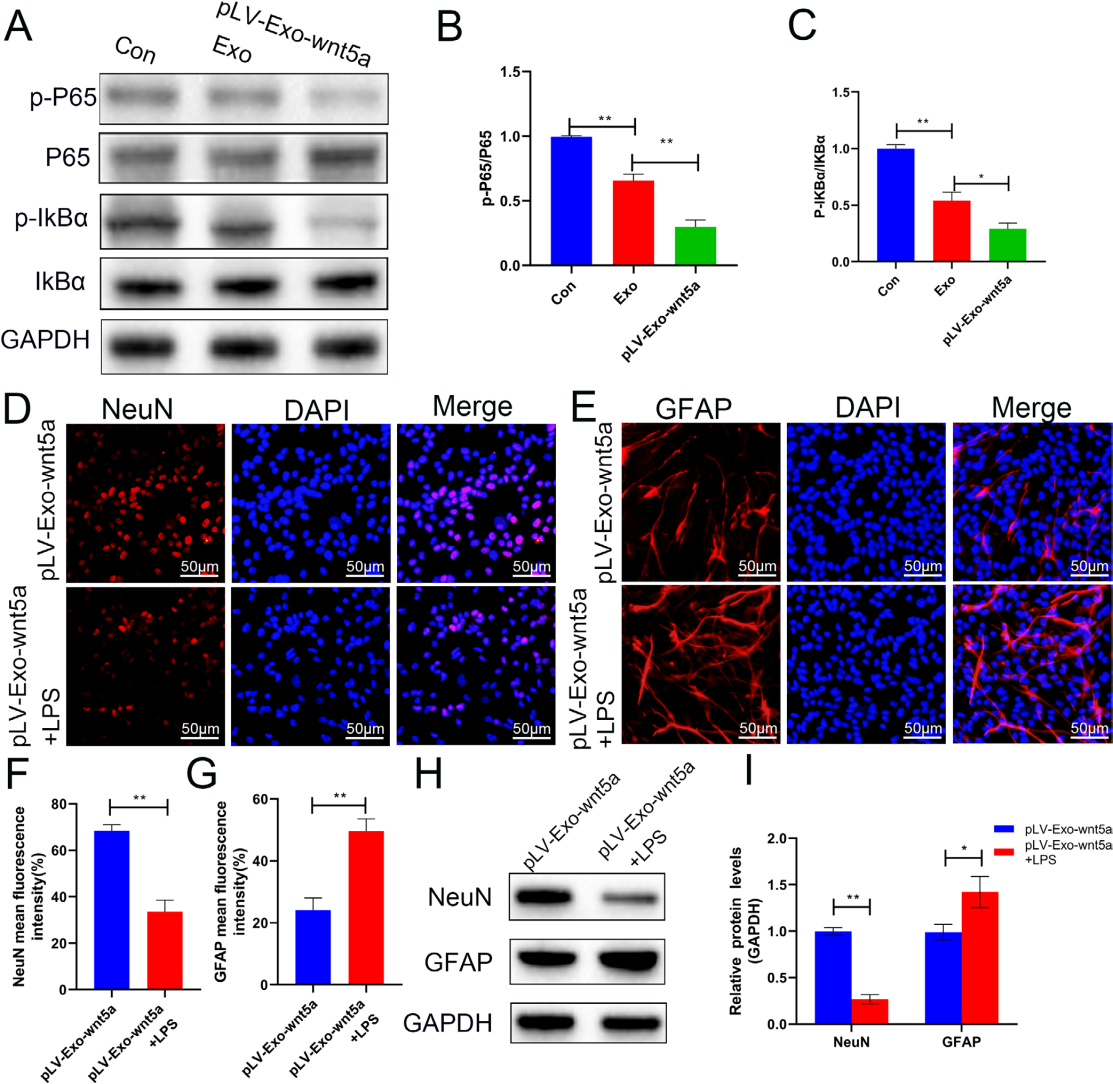
Wnt5a‐modified BMSC‐Exos affect neurons and astrocytes by inhibiting the NF‐κB signaling pathway. (A–C) The expression of P65 and IκBα and their phosphorylation in cells from the Con, Exo, and pLV‐Exo‐Wnt5a groups were detected by Western blot. (D–G) After intervention with the LPS to activate the NF‐κB pathway, the expression levels of NeuN and GFAP were determined using immunofluorescence (without LPS as a control). (H, I) After intervention with the LPS, the expression levels of NeuN and GFAP were determined using Western blot (without LPS as a control). Data are presented as mean ± SEM, *p < 0.05, **p < 0.01 compared to the adjacent group.

To investigate the role of the NF‐κB signaling pathway in the enhancement of neuronal differentiation of NSCs and suppression of astrocyte formation by BMSC‐Exos, we first assessed the expression and phosphorylation levels of P65 and IκBα in cells from the Con, Exo, and pLV‐Exo‐Wnt5a groups. The ratios of p‐P65 to total P65 and p‐IκBα to total IκBα were significantly reduced in the Exo and pLV‐Exo‐Wnt5a groups in contrast with the Con group (*p* < 0.05), with the lowest levels observed in the pLV‐Exo‐Wnt5a group (*p* < 0.05) (Figure 6A–C). Upon treatment with lipopolysaccharide (LPS) to activate the NF‐κB pathway, the fluorescence intensity of NeuN significantly decreased while GFAP intensity increased in the pLV‐Exo‐Wnt5a group (*p* < 0.05) (Figure 6D–G). WB analysis demonstrated consistent results (Figure 6H,I). The findings indicate that BMSC‐Exos promote neuronal differentiation of NSCs and suppress astrocyte formation through inhibition of the NF‐κB pathway, an effect enhanced by Wnt5a.

### 3.6 Wnt5a‐Modified BMSC‐Exos Promotes the Polarization of Microglia Towards the M2 Phenotype

**FIGURE 7:**
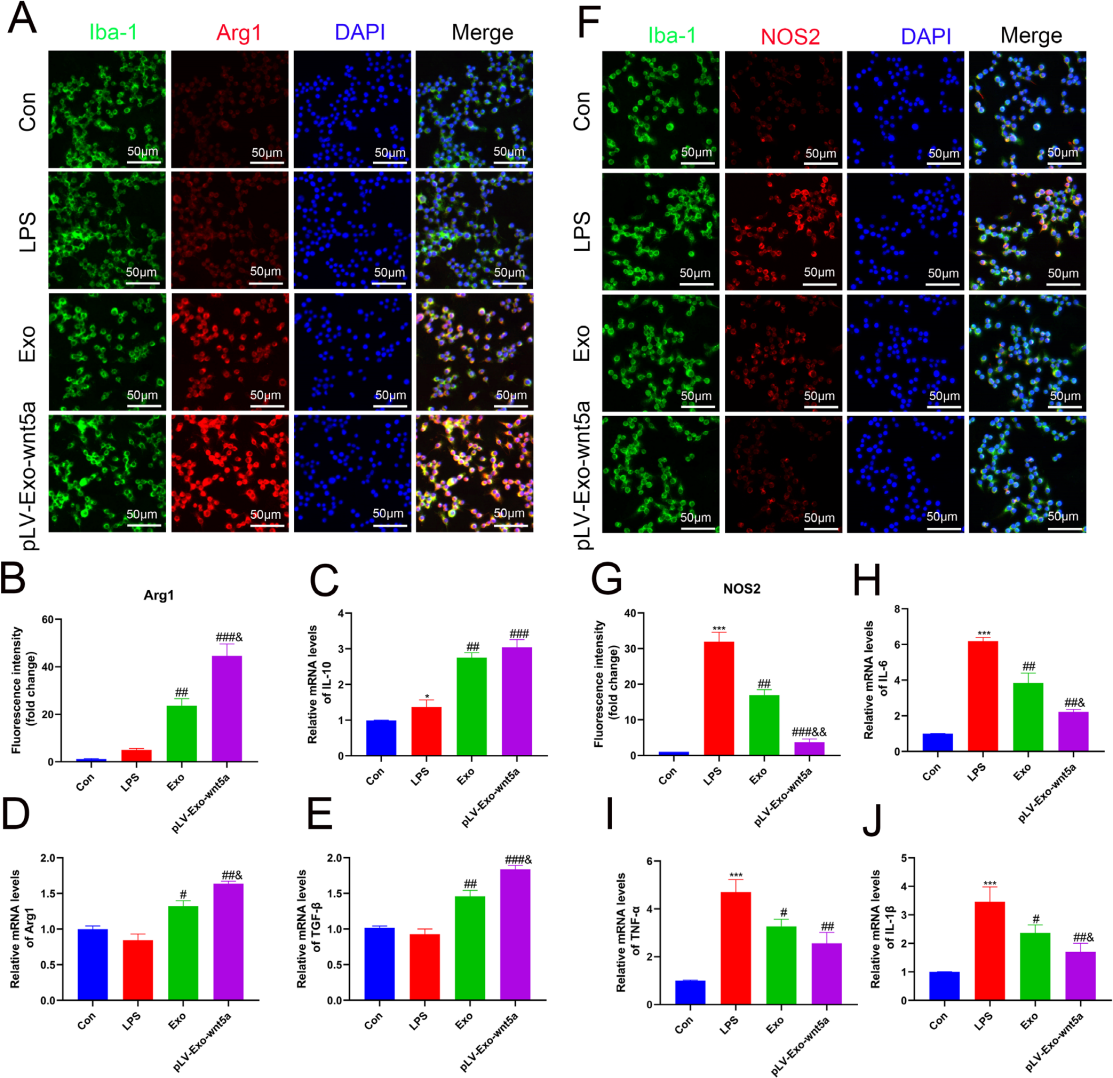
Wnt5a‐modified BMSC‐exo promotes microglial polarization towards the M2 phenotype. (A, B) Immunofluorescence staining was performed to detect the expression levels of microglia and M2 phenotype markers in the Con, LPS, Exo, and pLV‐Exo‐Wnt5a groups. (C–E) The mRNA levels of M2 phenotype markers in each group were determined by RT‐qPCR. (F, G) Immunofluorescence staining was performed to detect the expression levels of microglia and M1 phenotype markers in the Con, LPS, Exo, and pLV‐Exo‐Wnt5a groups. (H–J) The mRNA levels of M1 phenotype markers in each group were determined by RT‐qPCR. Data are presented as mean ± SEM, *p < 0.05, ***p < 0.001 compared to the Con group, #*p* < 0.05, ##*p* < 0.01, ###*p* < 0.001 compared to the LPS group, &*p* < 0.05, &&*p* < 0.01 compared to the Exo group.

To further validate whether BMSC‐Exos and Wnt5a are involved in regulating the inflammatory response in SCI, their effects on microglia were investigated in vivo. As shown in Figure 7, after LPS induction, the fluorescence intensity or mRNA expression of M1 phenotype‐associated markers (NOS2, IL‐6, IL‐1β, TNF‐α) in microglia significantly increased (*p* < 0.05), while no significant changes were observed in M2 phenotype‐associated markers (Arg1, IL‐10, TGF‐β). Following treatment with BMSC‐Exos, both the fluorescence intensity and mRNA expression of M1 phenotype markers were significantly downregulated, whereas those of M2 phenotype markers were significantly upregulated (*p* < 0.05). This conversion was further enhanced by Wnt5a modification (*p* < 0.05). These results indicate that Wnt5a‐modified BMSC‐Exos can promote the polarization of microglia towards the M2 phenotype and inhibit microglia‐derived inflammation during SCI.

### 3.7 Transplantation of Wnt5a‐Modified BMSC‐Exos Promoted Spinal Cord Tissue Repair in SCI Rats

**FIGURE 8:**
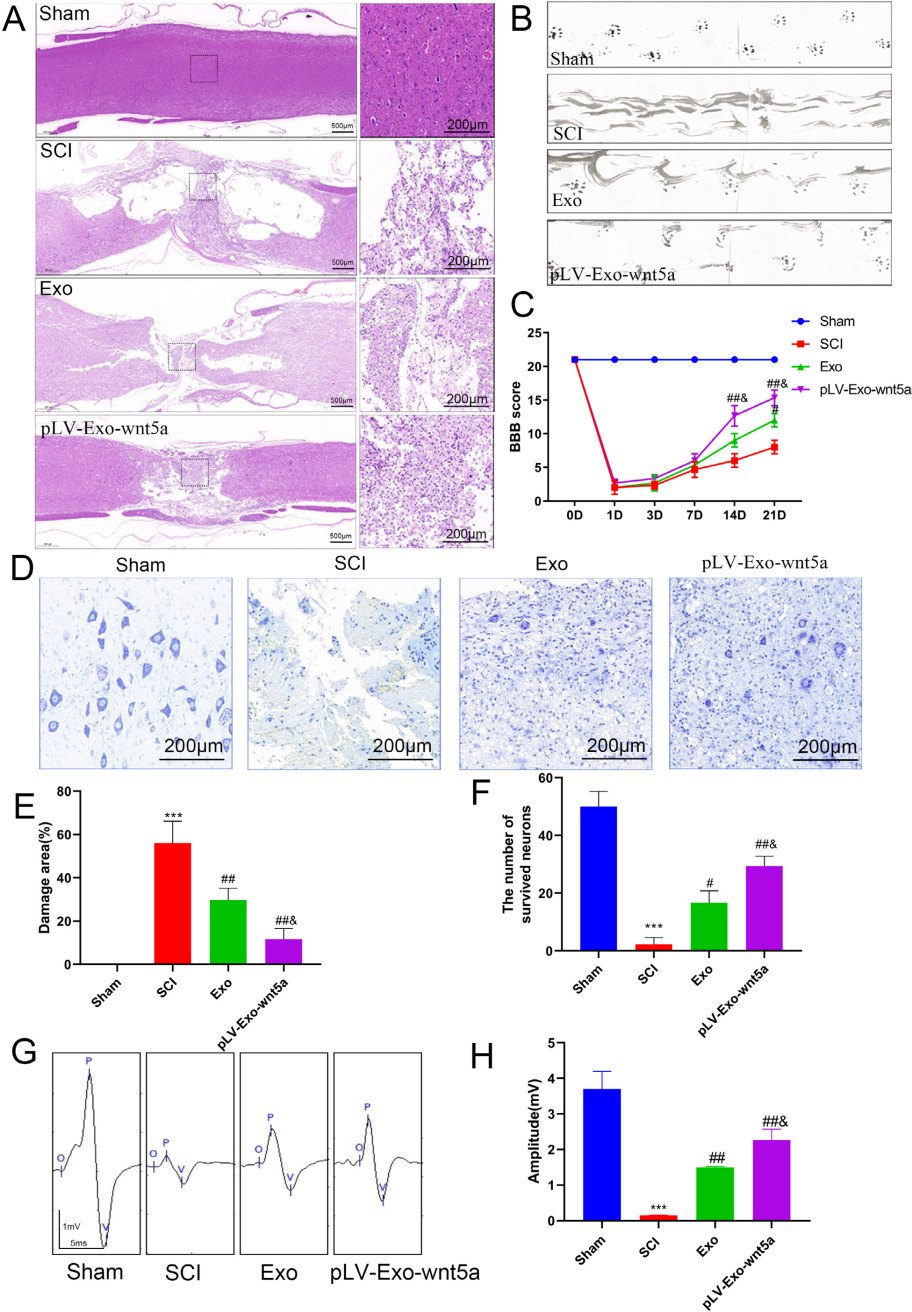
Transplantation of Wnt5a‐modified BMSC‐Exos promotes the recovery of spinal cord tissue and hindlimb function in rats with SCI. (A) HE staining results of spinal cord tissue in SCI rats. (B) Evaluation of changes in hindlimb motor function in SCI rats using the footprint test. (C) Motor function scores for each group of rats. (D) Nissl staining results of spinal cord tissue in SCI rats. (E) Percentage of injury area in spinal cord tissue for each group. (F) Percentage of surviving neurons in spinal cord tissue for each group. (G) Electrophysiological testing was performed on SCI rats from each group on day 21. (H) Comparison of MEP amplitudes among the different groups. Data are presented as mean ± SEM, *p < 0.05, ***p < 0.001 compared to the Sham group, #*p* < 0.05, ##*p* < 0.01 compared to the SCI group, &*p* < 0.05 compared to the Exo group.

To examine whether Wnt5a could facilitate the regeneration of spinal cord tissue by BMSC‐Exos in vivo, we examined histopathological changes in spinal cord tissue from SCI rats. Histopathological evaluation of spinal cord injury was conducted through HE staining to characterize tissue alterations. In contrast to sham‐operated controls, SCI‐induced lesions demonstrated marked structural disorganization with evident cystic cavitation. Notably, BMSC‐Exos administration remarkably reduced the pathological lesion area, accompanied by preserved tissue architecture (*p* < 0.05). Transplantation of Wnt5a‐overexpressing BMSC‐Exos further improved spinal cord tissue integrity and increased the number of normal cells (*p* < 0.05) (Figure 8A,E). Nissl staining results also supported these observations, with a significant increase in Nissl bodies following the transplantation of BMSC‐Exos (*p* < 0.05), which was further enhanced by the overexpression of Wnt5a (*p* < 0.05) (Figure 8D,F). Based on these results, it could be inferred that Wnt5a enhances the regenerative potential of BMSC‐Exos in injured spinal cord tissue.

### 3.8 Transplantation of Wnt5a‐Modified BMSC‐Exos Promoted the Recovery of Hindlimb Function in SCI Rats In Vivo

To further assess the impact on spinal cord recovery, we evaluated hindlimb function in rats. The footprint test revealed normal gait in the Sham group, while the SCI group showed dragging footprints. Partial improvement in right hindlimb gait was observed after transplantation of BMSC‐Exos, with a more obvious recovery among the Wnt5a‐modified BMSC‐Exos group (Figure 8B). These findings were supported by the BBB locomotor scale scores, which showed a significant improvement in both the Exo and pLV‐Exo‐Wnt5a groups in contrast with the SCI group on Days 14 and 21, with a more notable effect in the pLV‐Exo‐Wnt5a group (*p* < 0.05) (Figure 8C). These results suggest that Wnt5a‐modified BMSC‐Exos can promote functional recovery of hindlimb movement in vivo in animal models. The electrophysiological testing results further support the above conclusions. A significant increase in amplitude was observed in both the Exo group and the pLV‐Exo‐Wnt5a group, with the electrophysiological functional recovery in the pLV‐Exo‐Wnt5a group being superior to that in the Exo group (Figure 8G,H).

### 3.9 Transplantation of Wnt5a‐Modified BMSC‐Exos Modulated Microglial Phenotypes in Rat Spinal Cord Tissue

**FIGURE 9:**
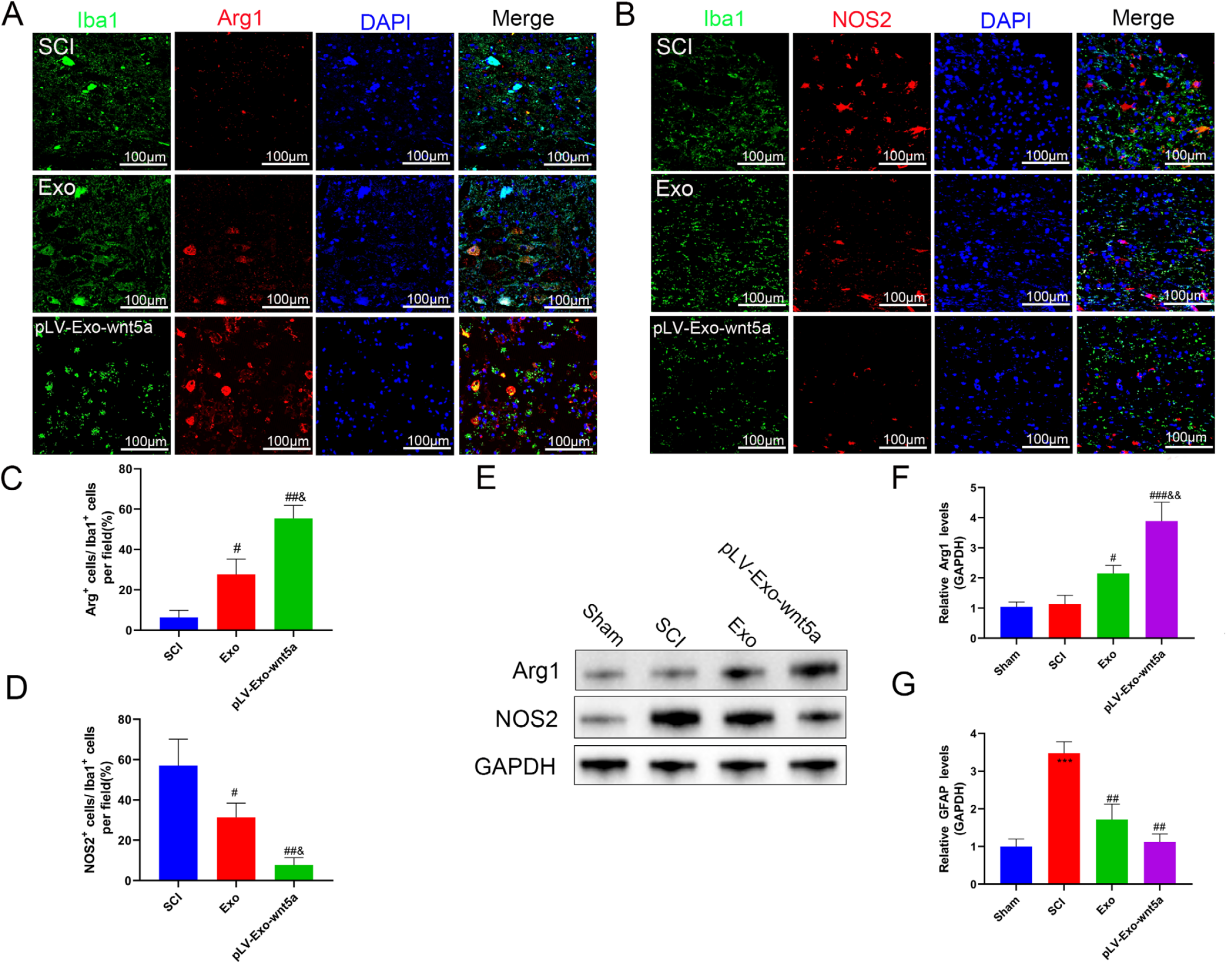
Transplantation of Wnt5a‐modified BMSC‐Exos promotes the modulation of microglial phenotypes in rat spinal cord tissue in vivo. (A–D) Relative expression levels of Iba1, Arg1, and NOS2 were determined using immunofluorescence assay. (E–G) Expression levels of Arg1 and NOS2 were measured by Western blot. Data are presented as mean ± SEM, *p < 0.05, ***p < 0.001 compared to the Sham group, #*p* < 0.05, ##*p* < 0.01, ###*p* < 0.001 compared to the SCI group, &*p* < 0.05, &&*p* < 0.01 compared to the Exo group.

Microglia play a pivotal role in SCI pathology by mediating inflammatory responses that exacerbate secondary damage. Iba1 serves as a marker for microglia, NOS2 indicates M1 polarization, and Arg1 signifies M2 polarization. In this study, immunofluorescence staining revealed that, compared to the SCI group, both the Exo and pLV‐Exo‐Wnt5a groups showed a higher percentage of Iba1^+^/Arg1^+^ cells (*p* < 0.05) and a lower percentage of Iba1^+^/NOS2^+^ cells (*p* < 0.05), with the most significant changes observed in the pLV‐Exo‐Wnt5a group (*p* < 0.05) (Figure 9A–D). Furthermore, the pLV‐Exo‐Wnt5a group showed increased Arg1 protein levels and decreased NOS2 protein levels in contrast with the Exo group (*p* < 0.05) (Figure 9E–G). These findings suggest that Wnt5a enhances the ability of BMSC‐Exos to modulate the anti‐inflammatory/pro‐inflammatory phenotype ratio in microglia after SCI, promoting their polarization towards the M2 phenotype.

### 3.10 Transplantation of Wnt5a‐Modified BMSC‐Exos Promoted Neuronal Regeneration and Reduced Astrocyte Proliferation In Vivo

**FIGURE 10:**
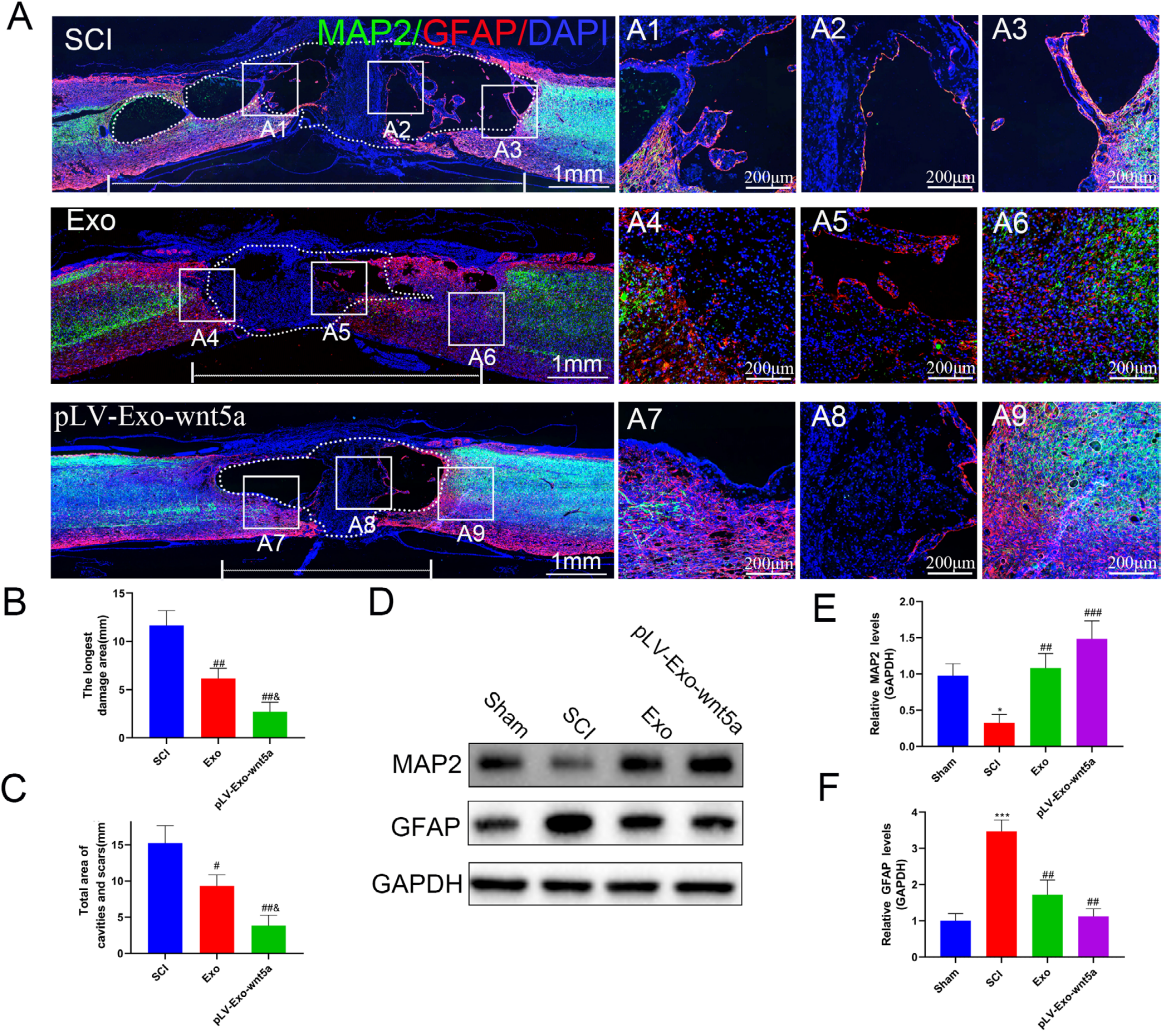
Wnt5a‐modified BMSC‐Exos reduce astrocyte production and increase mature neurons. (A) Immunofluorescence staining was used to observe MAP2 and GFAP‐positive cells in spinal cord tissues from different groups. (B) The longest distance of the injury area was quantified by immunofluorescence. (C) The area of the injury region was quantified by immunofluorescence. (D–F) The expression levels of MAP2 and GFAP in spinal cord tissues from different groups were determined by Western blot. Data are presented as mean ± SEM, *p < 0.05, ***p < 0.001 compared to the Sham group, #*p* < 0.05, ##*p* < 0.01, ###*p* < 0.001 compared to the SCI group, &*p* < 0.05 compared to the Exo group.

**FIGURE 11:**
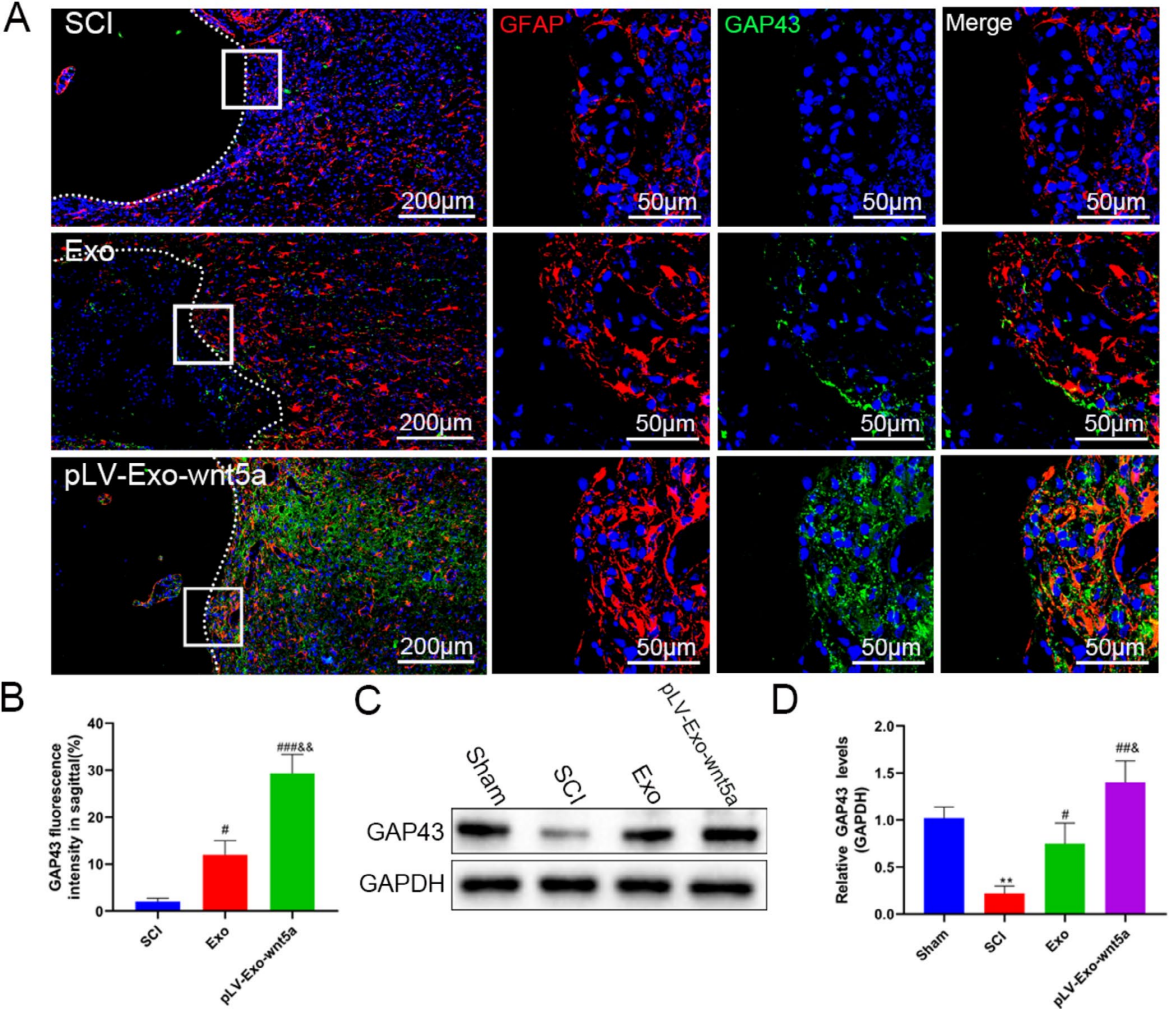
Wnt5a‐modified BMSC‐Exos promote neuron axon regeneration. (A, B) GAP43‐positive axons in spinal cord tissues of different groups were observed by immunofluorescence staining. (C, D) The expression levels of GAP43 in spinal cord tissues of different groups were determined by Western blot. Data are presented as mean ± SEM. *p < 0.05, **p < 0.01 compared to the Sham group; #*p* < 0.05, ##*p* < 0.01 compared to the SCI group; &*p* < 0.05, &&*p* < 0.01 compared to the Exo group.

To delve deeper into the in vivo effects of Wnt5a‐modified BMSC‐Exos on astrocytes and neurons, the length and area of SCI tissue were measured. Additionally, GFAP, a marker of mature astrocytes, and MAP2, a marker of neurons, were assessed. Fluorescence staining revealed that following the transplantation of BMSC‐Exos, both the length and area of the damaged spinal cord tissue were significantly reduced, accompanied by a significant decrease in the area of glial scars. Furthermore, the boundary of glial scars, formed by activated astrocytes, was less distinct in the stained regions (*p* < 0.05) (Figure 10A–C). Wnt5a modification further improved the condition of spinal cord tissue, as evidenced by the infiltration of the GFAP‐stained area into the MAP2‐stained region. WB analysis supported these findings (Figure 10D–F). Further analysis of the neuronal axon marker GAP43 showed a significant reduction in GAP43‐positive axons after surgery (*p* < 0.05), which was reversed by BMSC‐Exos transplantation, and further enhanced by Wnt5a modification (*p* < 0.05) (Figure 11A,B). These findings were further validated by WB analysis (Figure 11C,D). Collectively, these results suggest that BMSC‐Exos inhibit glial scar formation by astrocytes and promote the regeneration of neuron axons, with Wnt5a modification enhancing these effects.

## 4 Discussion

SCI is a neurological condition that significantly impacts both patients and their families [34]. SCI is classified into two distinct phases: primary injury and subsequent injury. The primary injury phase involves irreversible spinal cord damage due to direct or indirect trauma [35]. The subsequent injury phase, occurring within minutes to days after the initial trauma and potentially lasting for up to a month, is marked by pathological changes, including neuroinflammation, oxidative stress, and cell apoptosis. These changes further compromise the tissue microenvironment, thereby impeding neurogenesis and axonal regeneration and contributing to the limited therapeutic effectiveness in SCI [2, 36].

With advances in regenerative medicine, MSC transplantation has become a potential approach for neural repair in SCI [37, 38]. However, the efficacy of MSC transplantation remains inconsistent, often accompanied by various side effects. Recent research increasingly suggests that paracrine signaling may be critical for the effects of MSCs [10]. Exos, considering key mediators of this paracrine signaling, possess biological functions similar to those of MSCs. They contain a variety of trophic and growth factors, offering advantages such as stability, ease of isolation, and the absence of immune response activation [39]. Numerous studies have demonstrated that MSC‐Exos can have comparable or even superior therapeutic effects in various diseases [40, 41, 42, 43]. Furthermore, due to their small size and membrane permeability, Exos can efficiently reach damaged regions of the nervous system, overcoming the challenge posed by the blood‐spinal cord barrier during MSC homing [44, 45]. Therefore, the transplantation of MSC‐Exos presents unique advantages for SCI repair [46, 47].

The Wnt signaling pathway is widely conserved in multicellular eukaryotes and plays a critical role in regulating key biological processes through its complex signaling mechanisms. It is essential for normal development, tissue homeostasis, and organ function. Depending on the downstream signaling pathway, the Wnt family of proteins exerts diverse functions through at least three distinct cascades: the canonical Wnt/β‐catenin pathway, the non‐canonical Wnt/Ca^2+^ pathway, and the Wnt/Planar Cell Polarity (PCP) pathway [48, 49]. Wnt5a is a ligand for the non‐canonical Wnt signaling pathway with various biological functions. Our team has found that Wnt5a exerts a powerful influence on BMSCs [26]. It drives BMSCs towards neuronal differentiation into neurons while reducing the formation of astrocytes. Based on these findings, our study demonstrates that BMSC‐Exos also stimulate neuronal proliferation and reduce astrocyte production, with Wnt5a induction leading to even more favorable outcomes.

Inflammation is a key pathological process in secondary SCI and serves as an essential defense mechanism that clears tissue debris and promotes wound healing. However, inflammatory cells produce toxic molecules that can damage otherwise healthy tissue, inhibit nerve regeneration, and impede the efficacy of subsequent SCI treatments [50]. Previous studies have indicated that both stem cells and MSC‐Exos can modulate inflammation following SCI by releasing cytokines and chemokines [51, 52]. However, the regulatory role of Wnt5a in this process remains unclear. Through high‐throughput RNA sequencing and KEGG pathway enrichment analysis, our study provides evidence linking Wnt5a‐mediated BMSC‐Exos treatment in SCI to inflammation, specifically through the NF‐κB signaling pathway. By analyzing the phosphorylation of P65 and IκBα and intervening with the LPS to activate the NF‐κB pathway, we demonstrated that the therapeutic effects of Wnt5a are associated with the inhibition of the NF‐κB pathway. Traditionally, Wnt5a has been thought to activate the NF‐κB signaling pathway, playing a stimulatory role in inflammation. However, research by Li et al. [53] suggests that TNF‐α‐induced Wnt5a could inhibit NF‐κB signaling by activating the JNK‐AP1 (JunB)‐Sox9 axis, thereby antagonizing the TNF‐α‐induced upregulation of matrix metalloproteinases, forming a negative feedback loop. Studies by Elizabeth et al. also indicate a possible antagonistic relationship between NFAT/Sox9 downstream of Wnt5a and NF‐κB signaling [54]. These findings were consistent with our results, indicating that Wnt5a may regulate immune responses by inhibiting the NF‐κB signaling pathway, thereby reducing neuroinflammation after SCI. This ultimately promotes neuronal differentiation of NSCs induced by BMSC‐Exos while reducing astrocyte production.

To demonstrate the efficacy of Wnt5a‐modified Exos in vivo, animal experiments were conducted. Both rat locomotor function tests and histopathological findings revealed that Wnt5a‐modified BMSC‐Exos can effectively repair spinal cord tissue and restore hindlimb motor function in rats. The findings were further substantiated through the detection of specific biomarkers that are characteristic of neurons and astrocytes within spinal cord tissue. The scars formed by astrocytes serve as barriers to neuronal regeneration. Previous studies have shown that MSC‐Exos reduce the percentage of astrocytes following SCI by suppressing NF‐κB activation, consistent with our findings [55]. To further explore the immune regulation of Wnt5a, we examined microglia. Microglia are crucial immune cells in the brain and spinal cord. Their activation was associated with the release of proinflammatory mediators, making them key initiators of sustained inflammatory responses [56, 57]. Microglia exist in two phenotypes: proinflammatory (M1) and anti‐inflammatory (M2). The phenotypic shift between these states determines the course of inflammation [58]. Previous research indicates that the effects of Wnt5a on glial cells vary based on biological context and are influenced by multiple factors [59]. The study suggests that Wnt5a accelerates the proinflammatory response of microglia [60], while Carina et al. have demonstrated that Wnt5a has dual proinflammatory and anti‐inflammatory effects on microglia [61]. Furthermore, Exos themselves can influence the phenotypic changes of microglia. Liu et al. [19] reported that Exo administration promoted M2 polarization of microglia and generated anti‐inflammatory factors both in vitro and in vivo. This finding was corroborated by another study on Exo‐induced macrophage M2 polarization [20]. Our study demonstrates for the first time that BMSC‐Exos overexpressing Wnt5a promote M2 polarization of microglia and enhance anti‐inflammatory effects. This has not been reported previously. The reconstruction of neural pathways is a prerequisite for functional recovery, and the glial scar formed by astrocytes after SCI serves as a critical factor that hinders the formation of nerve axons and the reconstruction of neural pathways [62, 63]. Previous studies have indicated that M1 microglia are predominantly located within the fibrotic component of glial scars, possibly due to the association between the inflammatory environment and glial scar formation. Modulating microglia polarization towards the M2 phenotype can effectively inhibit glial scar formation, corroborating our findings [64].

In summary, this study is the first to explore neuronal differentiation of NSCs induced by Wnt5a‐modified BMSC‐Exos for functional recovery after SCI. Through both in vitro and in vivo experiments, we documented 4 key observations, providing insights into Wnt5a‐induced neuronal differentiation. Firstly, this study confirmed that BMSC‐Exos could promote proliferation of NSCs, neuronal differentiation, and reduction of astrocytes, with Wnt5a induction achieving the most favorable results. Secondly, transcriptome sequencing analysis revealed a correlation between Wnt5a and the NF‐κB signaling pathway, which was verified through in vitro experiments. Thirdly, research on spinal cord tissue demonstrated that BMSC‐Exos overexpressing Wnt5a promoted M2 polarization of microglia and enhanced anti‐inflammatory effects. Finally, we provided in vivo evidence showing that Wnt5a‐induced BMSC‐Exos promoted neuronal differentiation of NSCs, reduced astrocyte presence, and improved SCI‐related symptoms within animal models. These results suggest the promising application of BMSC‐Exos in the treatment of SCI.

## 5 Conclusion

This study demonstrates that Wnt5a positively influences neuronal regeneration after SCI via BMSC‐Exos, potentially through immune regulation and suppression of neuroinflammation. However, additional research is essential to uncover the intricate mechanisms underlying these phenomena. Our results also suggest that transplantation of Wnt5a‐modified BMSC‐Exos could enhance tissue repair and the recovery of motor function after SCI in vivo, highlighting their potential in treating SCI.

## Author Contributions

D.L., E.F., Y.W., Z.H., and C.L. co‐conducted the in vitro and in vivo experiments, D.L. and C.L. authored the manuscript; L.C. and C.Y. conducted animal grouping and data analysis; S.L., L.M., and K.N. co‐edited the figures; D.G., B.Z., and D.L. co‐designed the experiments, with X.L. providing guidance on the research protocol and funding support for the study; X.L., D.L., and C.L. co‐proofread the manuscript, and all authors reviewed the final draft.

## Funding

This work was supported by Natural Science Foundation of Guangdong, China (Nos. 2024A1515030293, 2022A1515010793, 2024A1515140021); Research Fund for Bajian/Qingmiao Talents of Guangdong Provincial Hospital of Chinese Medicine (Nos. BJ2022KY07, SZ2022QN05); University—Hospital Joint Fund Project of Guangzhou University of Chinese Medicine (Nos. GZYZS2024D03, GZY2025GB0402); Administration of Traditional Chinese Medicine of Guangdong Province, China (No. 20241126).

## Disclosure

The authors declare that they have not used AI‐generated work in this manuscript.

ARRIVE checklist: The work has been reported in line with the ARRIVE guidelines 2.0.

## Ethics Statement

The animal experiment was complied with the Compilation of Group Standards and Implementation Guidelines of the Chinese Society for Laboratory Animals (CSLA). The animal care procedures were reviewed and approved by the Animal Ethics Committee of Guangzhou University of Chinese Medicine (Title of the approved project: The Mechanism of Wnt5a‐Overexpressed BMSCs Promote Spinal Cord Injury Repairing based on Cross‐Talk Between MAPK/JNK and Notch Signaling Pathway and the Treatment of Bushen Huoxue Decoction after Spinal Cord Injury, Approval number: 2023013, Approval date: 2023.02.20).

## Consent

We obtained permissions from the participants to publish their data. All participants gave written consent for publication.

## Conflicts of Interest

The authors declare no conflicts of interest.

## Supporting information


**Data S1:** cns70834‐sup‐0001‐supinfo.doc.

## Data Availability

The data used to support the findings of this study are available from the corresponding authors upon request. RNA‐Seq data are deposited in the NCBl Gene Expression Omnibus (GEO) repository at https://www.ncbi.nlm.nih.gov/geo/ under accessions GSE296458.

## References

[1] A. Alizadeh , S. M. Dyck , and S. Karimi‐Abdolrezaee , “Traumatic Spinal Cord Injury: An Overview of Pathophysiology, Models and Acute Injury Mechanisms,” Frontiers in Neurology 10 (2019): 282, 10.3389/fneur.2019.00282. 30967837 PMC6439316

[2] X. Hu , W. Xu , Y. Ren , et al., “Spinal Cord Injury: Molecular Mechanisms and Therapeutic Interventions,” Signal Transduction and Targeted Therapy 8 (2023): 245, 10.1038/s41392-023-01477-6. 37357239 PMC10291001

[3] B. Fan , Z. Wei , X. Yao , et al., “Microenvironment Imbalance of Spinal Cord Injury,” Cell Transplantation 27 (2018): 853–866, 10.1177/0963689718755778. 29871522 PMC6050904

[4] S. M. Hosseini , B. Borys , and S. Karimi‐Abdolrezaee , “Neural Stem Cell Therapies for Spinal Cord Injury Repair: An Update on Recent Preclinical and Clinical Advances,” Brain 147 (2024): 766–793, 10.1093/brain/awad392. 37975820

[5] N. Li and J. Hua , “Interactions Between Mesenchymal Stem Cells and the Immune System,” Cellular and Molecular Life Sciences 74 (2017): 2345–2360, 10.1007/s00018-017-2473-5. 28214990 PMC11107583

[6] J. Li , W. Luo , C. Xiao , et al., “Recent Advances in Endogenous Neural Stem/Progenitor Cell Manipulation for Spinal Cord Injury Repair,” Theranostics 13 (2023): 3966–3987, 10.7150/thno.84133. 37554275 PMC10405838

[7] W. Zakrzewski , M. Dobrzyński , M. Szymonowicz , and Z. Rybak , “Stem Cells: Past, Present, and Future,” Stem Cell Research & Therapy 10 (2019): 68, 10.1186/s13287-019-1165-5. 30808416 PMC6390367

[8] C. Toma , W. R. Wagner , S. Bowry , A. Schwartz , and F. Villanueva , “Fate of Culture‐Expanded Mesenchymal Stem Cells in the Microvasculature: In Vivo Observations of Cell Kinetics,” Circulation Research 104 (2009): 398–402, 10.1161/CIRCRESAHA.108.187724. 19096027 PMC3700384

[9] E. Eggenhofer , V. Benseler , A. Kroemer , et al., “Mesenchymal Stem Cells Are Short‐Lived and Do Not Migrate Beyond the Lungs After Intravenous Infusion,” Frontiers in Immunology 3 (2012): 297, 10.3389/fimmu.2012.00297. 23056000 PMC3458305

[10] F. Vizoso , N. Eiro , S. Cid , J. Schneider , and R. Perez‐Fernandez , “Mesenchymal Stem Cell Secretome: Toward Cell‐Free Therapeutic Strategies in Regenerative Medicine,” International Journal of Molecular Sciences 18 (2017): 1852, 10.3390/ijms18091852. 28841158 PMC5618501

[11] S. Pluchino and J. A. Smith , “Explicating Exosomes: Reclassifying the Rising Stars of Intercellular Communication,” Cell 177 (2019): 225–227, 10.1016/j.cell.2019.03.020. 30951665

[12] Y. Zhang , J. Bi , J. Huang , Y. Tang , S. Du , and P. Li , “Exosome: A Review of Its Classification, Isolation Techniques, Storage, Diagnostic and Targeted Therapy Applications,” International Journal of Nanomedicine 15 (2020): 6917–6934, 10.2147/IJN.S264498. 33061359 PMC7519827

[13] F. Tan , X. Li , Z. Wang , J. Li , K. Shahzad , and J. Zheng , “Clinical Applications of Stem Cell‐Derived Exosomes,” Signal Transduction and Targeted Therapy 9 (2024): 17, 10.1038/s41392-023-01704-0. 38212307 PMC10784577

[14] X. Sun , X. Peng , Y. Cao , Y. Zhou , and Y. Sun , “ADNP Promotes Neural Differentiation by Modulating Wnt/β‐Catenin Signaling,” Nature Communications 11 (2020): 2984, 10.1038/s41467-020-16799-0. PMC7293280 32533114

[15] S.‐Y. Park , M.‐J. Kang , and J.‐S. Han , “Interleukin‐1 Beta Promotes Neuronal Differentiation Through the Wnt5a/RhoA/JNK Pathway in Cortical Neural Precursor Cells,” Molecular Brain 11 (2018): 39, 10.1186/s13041-018-0383-6. 29973222 PMC6033214

[16] Y. Hou , C. Liang , L. Sui , et al., “Curculigoside Regulates Apoptosis and Oxidative Stress Against Spinal Cord Injury by Modulating the Nrf‐2/NQO‐1 Signaling Pathway In Vitro and In Vivo,” Molecular Neurobiology 62 (2025): 3082–3097, 10.1007/s12035-024-04409-9. 39230866 PMC11790752

[17] L. Urdzíková , J. Růžička , M. LaBagnara , et al., “Human Mesenchymal Stem Cells Modulate Inflammatory Cytokines After Spinal Cord Injury in Rat,” IJMS 15 (2014): 11275–11293, 10.3390/ijms150711275. 24968269 PMC4139782

[18] G. Sun , G. Li , D. Li , et al., “hucMSC Derived Exosomes Promote Functional Recovery in Spinal Cord Injury Mice via Attenuating Inflammation,” Materials Science and Engineering: C 89 (2018): 194–204, 10.1016/j.msec.2018.04.006. 29752089

[19] W. Liu , Y. Rong , J. Wang , et al., “Exosome‐Shuttled miR‐216a‐5p From Hypoxic Preconditioned Mesenchymal Stem Cells Repair Traumatic Spinal Cord Injury by Shifting Microglial M1/M2 Polarization,” Journal of Neuroinflammation 17 (2020): 47, 10.1186/s12974-020-1726-7. 32019561 PMC7001326

[20] Y. Nakao , T. Fukuda , Q. Zhang , et al., “Exosomes From TNF‐α‐Treated Human Gingiva‐Derived MSCs Enhance M2 Macrophage Polarization and Inhibit Periodontal Bone Loss,” Acta Biomaterialia 122 (2021): 306–324, 10.1016/j.actbio.2020.12.046. 33359765 PMC7897289

[21] T. Li , R. W. S. Chan , C.‐L. Lee , et al., “WNT5A Interacts With FZD5 and LRP5 to Regulate Proliferation and Self‐Renewal of Endometrial Mesenchymal Stem‐Like Cells,” Frontiers in Cell and Development Biology 10 (2022): 837827, 10.3389/fcell.2022.837827. PMC8919396 35295855

[22] B. J. Povinelli and M. J. Nemeth , “Wnt5a Regulates Hematopoietic Stem Cell Proliferation and Repopulation Through the Ryk Receptor,” Stem Cells 32 (2014): 105–115, 10.1002/stem.1513. 23939973 PMC5576736

[23] W. Liu , L. Du , Y. Cui , C. He , and Z. He , “WNT5A Regulates the Proliferation, Apoptosis and Stemness of Human Stem Leydig Cells via the β‐Catenin Signaling Pathway,” Cellular and Molecular Life Sciences 81 (2024): 93, 10.1007/s00018-023-05077-z. 38367191 PMC11072989

[24] L. Liu , S. Luo , Q. Li , et al., “Role of Wnt5a in Modulation of Osteoporotic Adipose‐Derived Stem Cells and Osteogenesis,” Cell Proliferation 58 (2025): e13747, 10.1111/cpr.13747. 39288944 PMC11839189

[25] X. Li , Z. Peng , L. Long , et al., “Transplantation of Wnt5a‐Modified NSCs Promotes Tissue Repair and Locomotor Functional Recovery After Spinal Cord Injury,” Experimental & Molecular Medicine 52 (2020): 2020–2033, 10.1038/s12276-020-00536-0. 33311637 PMC8080632

[26] H. Yang , C. Liang , J. Luo , et al., “Transplantation of Wnt5a‐Modified Bone Marrow Mesenchymal Stem Cells Promotes Recovery After Spinal Cord Injury via the PI3K/AKT Pathway,” Molecular Neurobiology 61 (2024): 10830–10844, 10.1007/s12035-024-04248-8. 38795301 PMC11584464

[27] L. F. Geffner , P. Santacruz , M. Izurieta , et al., “Administration of Autologous Bone Marrow Stem Cells Into Spinal Cord Injury Patients via Multiple Routes Is Safe and Improves Their Quality of Life: Comprehensive Case Studies,” Cell Transplantation 17 (2008): 1277–1293, 10.3727/096368908787648074. 19364066

[28] Y. Wang , T. Zhao , Y. Jiao , et al., “Silicate Nanoplatelets Promotes Neuronal Differentiation of Neural Stem Cells and Restoration of Spinal Cord Injury,” Advanced Healthcare Materials 12 (2023): 2203051, 10.1002/adhm.202203051. 37141006

[29] Z. Fan , M. Jia , J. Zhou , et al., “Pharmacological Targeting cGAS/STING/NF‐κB Axis by Tryptanthrin Induces Microglia Polarization Toward M2 Phenotype and Promotes Functional Recovery in a Mouse Model of Spinal Cord Injury,” Neural Regeneration Research 20 (2025): 3287–3301, 10.4103/NRR.NRR-D-23-01256. 38993129 PMC11881704

[30] Y. Li , N. Khan , R. M. Ritzel , et al., “Sexually Dimorphic Extracellular Vesicle Responses After Chronic Spinal Cord Injury Are Associated With Neuroinflammation and Neurodegeneration in the Aged Brain,” Journal of Neuroinflammation 20 (2023): 197, 10.1186/s12974-023-02881-z. 37653491 PMC10469550

[31] D. M. Basso , M. S. Beattie , and J. C. Bresnahan , “A Sensitive and Reliable Locomotor Rating Scale for Open Field Testing in Rats,” Journal of Neurotrauma 12 (1995): 1–21, 10.1089/neu.1995.12.1. 7783230

[32] A. S. Rivlin and C. H. Tator , “Objective Clinical Assessment of Motor Function After Experimental Spinal Cord Injury in the Rat,” Journal of Neurosurgery 47 (1977): 577–581, 10.3171/jns.1977.47.4.0577. 903810

[33] Z. Li , Y. Qi , L. Sun , et al., “Three‐Dimensional Nanofibrous Sponges With Aligned Architecture and Controlled Hierarchy Regulate Neural Stem Cell Fate for Spinal Cord Regeneration,” Theranostics 13 (2023): 4762–4780, 10.7150/thno.87288. 37771775 PMC10526661

[34] G. Courtine and M. V. Sofroniew , “Spinal Cord Repair: Advances in Biology and Technology,” Nature Medicine 25 (2019): 898–908, 10.1038/s41591-019-0475-6. 31160817

[35] H. Cowan , C. Lakra , and M. Desai , “Autonomic Dysreflexia in Spinal Cord Injury,” BMJ 371 (2020): m3596, 10.1136/bmj.m3596. 33008797

[36] A. Anjum , M. D. Yazid , M. Fauzi Daud , et al., “Spinal Cord Injury: Pathophysiology, Multimolecular Interactions, and Underlying Recovery Mechanisms,” International Journal of Molecular Sciences 21 (2020): 7533, 10.3390/ijms21207533. 33066029 PMC7589539

[37] G. Schepici , S. Silvestro , and E. Mazzon , “Regenerative Effects of Exosomes‐Derived MSCs: An Overview on Spinal Cord Injury Experimental Studies,” Biomedicine 11 (2023): 201, 10.3390/biomedicines11010201. PMC9855467 36672709

[38] H. Zamani , M. Soufizomorrod , S. Oraee‐Yazdani , et al., “Safety and Feasibility of Autologous Olfactory Ensheathing Cell and Bone Marrow Mesenchymal Stem Cell Co‐Transplantation in Chronic Human Spinal Cord Injury: A Clinical Trial,” Spinal Cord 60 (2022): 63–70, 10.1038/s41393-021-00687-5. 34504283

[39] D. Li , D. Li , Z. Wang , et al., “Signaling Pathways Activated and Regulated by Stem Cell‐Derived Exosome Therapy,” Cell & Bioscience 14 (2024): 105, 10.1186/s13578-024-01277-7. 39164778 PMC11334359

[40] G. Lou , Z. Chen , M. Zheng , and Y. Liu , “Mesenchymal Stem Cell‐Derived Exosomes as a New Therapeutic Strategy for Liver Diseases,” Experimental & Molecular Medicine 49 (2017): e346, 10.1038/emm.2017.63. 28620221 PMC5519012

[41] W. Liu , Y. Wang , F. Gong , et al., “Exosomes Derived From Bone Mesenchymal Stem Cells Repair Traumatic Spinal Cord Injury by Suppressing the Activation of A1 Neurotoxic Reactive Astrocytes,” Journal of Neurotrauma 36 (2019): 469–484, 10.1089/neu.2018.5835. 29848167

[42] Y. Lu , M. Liu , X. Guo , et al., “miR‐26a‐5p Alleviates CFA‐Induced Chronic Inflammatory Hyperalgesia Through Wnt5a/CaMKII/NFAT Signaling in Mice,” CNS Neuroscience & Therapeutics 29 (2023): 1254–1271, 10.1111/cns.14099. 36756710 PMC10068476

[43] Z. Lin , Y. Wu , Y. Xu , G. Li , Z. Li , and T. Liu , “Mesenchymal Stem Cell‐Derived Exosomes in Cancer Therapy Resistance: Recent Advances and Therapeutic Potential,” Molecular Cancer 21 (2022): 179, 10.1186/s12943-022-01650-5. 36100944 PMC9468526

[44] Y. Xiong , A. Mahmood , and M. Chopp , “Emerging Potential of Exosomes for Treatment of Traumatic Brain Injury,” Neural Regeneration Research 12 (2017): 19–22, 10.4103/1673-5374.198966. 28250732 PMC5319225

[45] T. Hua , M. Yang , H. Song , et al., “Huc‐MSCs‐Derived Exosomes Attenuate Inflammatory Pain by Regulating Microglia Pyroptosis and Autophagy via the miR‐146a‐5p/TRAF6 Axis,” Journal of Nanobiotechnology 20 (2022): 324, 10.1186/s12951-022-01522-6. 35836229 PMC9281091

[46] P. Gao , J. Yi , W. Chen , et al., “Pericyte‐Derived Exosomal miR‐210 Improves Mitochondrial Function and Inhibits Lipid Peroxidation in Vascular Endothelial Cells After Traumatic Spinal Cord Injury by Activating JAK1/STAT3 Signaling Pathway,” Journal of Nanobiotechnology 21 (2023): 452, 10.1186/s12951-023-02110-y. 38012616 PMC10680350

[47] J. Ren , B. Zhu , G. Gu , et al., “Schwann Cell‐Derived Exosomes Containing MFG‐E8 Modify Macrophage/Microglial Polarization for Attenuating Inflammation via the SOCS3/STAT3 Pathway After Spinal Cord Injury,” Cell Death & Disease 14 (2023): 70, 10.1038/s41419-023-05607-4. 36717543 PMC9887051

[48] K. Wang , F. Ma , S. Arai , et al., “WNT5a Signaling Through ROR2 Activates the Hippo Pathway to Suppress YAP1 Activity and Tumor Growth,” Cancer Research 83 (2023): 1016–1030, 10.1158/0008-5472.CAN-22-3003. 36622276 PMC10073315

[49] M. L. P. Bueno , S. T. O. Saad , and F. M. Roversi , “WNT5A in Tumor Development and Progression: A Comprehensive Review,” Biomedicine & Pharmacotherapy 155 (2022): 113599, 10.1016/j.biopha.2022.113599. 36089446

[50] D. J. Hellenbrand , C. M. Quinn , Z. J. Piper , C. N. Morehouse , J. A. Fixel , and A. S. Hanna , “Inflammation After Spinal Cord Injury: A Review of the Critical Timeline of Signaling Cues and Cellular Infiltration,” Journal of Neuroinflammation 18 (2021): 284, 10.1186/s12974-021-02337-2. 34876174 PMC8653609

[51] X. Freyermuth‐Trujillo , J. J. Segura‐Uribe , H. Salgado‐Ceballos , C. E. Orozco‐Barrios , and A. Coyoy‐Salgado , “Inflammation: A Target for Treatment in Spinal Cord Injury,” Cells 11 (2022): 2692, 10.3390/cells11172692. 36078099 PMC9454769

[52] X. Lu , G. Xu , Z. Lin , et al., “Engineered Exosomes Enriched in Netrin‐1 modRNA Promote Axonal Growth in Spinal Cord Injury by Attenuating Inflammation and Pyroptosis,” Biomaterials Research 27 (2023): 3, 10.1186/s40824-023-00339-0. 36647161 PMC9843879

[53] Z. Li , K. Zhang , X. Li , et al., “Wnt5a Suppresses Inflammation‐Driven Intervertebral Disc Degeneration via a TNF‐α/NF‐κB–Wnt5a Negative‐Feedback Loop,” Osteoarthritis and Cartilage 26 (2018): 966–977, 10.1016/j.joca.2018.04.002. 29656141

[54] E. W. Bradley and M. H. Drissi , “WNT5A Regulates Chondrocyte Differentiation Through Differential Use of the CaN/NFAT and IKK/NF‐κB Pathways,” Molecular Endocrinology 24 (2010): 1581–1593, 10.1210/me.2010-0037. 20573686 PMC5417459

[55] L. Wang , S. Pei , L. Han , et al., “Mesenchymal Stem Cell‐Derived Exosomes Reduce A1 Astrocytes via Downregulation of Phosphorylated NFκB P65 Subunit in Spinal Cord Injury,” Cellular Physiology and Biochemistry 50 (2018): 1535–1559, 10.1159/000494652. 30376671

[56] H. S. Kwon and S.‐H. Koh , “Neuroinflammation in Neurodegenerative Disorders: The Roles of Microglia and Astrocytes,” Translational Neurodegeneration 9 (2020): 42, 10.1186/s40035-020-00221-2. 33239064 PMC7689983

[57] K. Borst , A. A. Dumas , and M. Prinz , “Microglia: Immune and Non‐Immune Functions,” Immunity 54 (2021): 2194–2208, 10.1016/j.immuni.2021.09.014. 34644556

[58] H. Zhang , L. Xiang , H. Yuan , and H. Yu , “PTPRO Inhibition Ameliorates Spinal Cord Injury Through Shifting Microglial M1/M2 Polarization via the NF‐κB/STAT6 Signaling Pathway,” Biochimica et Biophysica Acta (BBA)—Molecular Basis of Disease 1870 (2024): 167141, 10.1016/j.bbadis.2024.167141. 38565385

[59] P. González , C. González‐Fernández , and F. Javier Rodríguez , “Effects of Wnt5a Overexpression in Spinal Cord Injury,” Journal of Cellular and Molecular Medicine 25 (2021): 5150–5163, 10.1111/jcmm.16507. 33939286 PMC8178287

[60] S. Yuan , Y. Shi , K. Guo , and S.‐J. Tang , “Nucleoside Reverse Transcriptase Inhibitors (NRTIs) Induce Pathological Pain Through Wnt5a‐Mediated Neuroinflammation in Aging Mice,” Journal of Neuroimmune Pharmacology 13 (2018): 230–236, 10.1007/s11481-018-9777-6. 29429030 PMC5930064

[61] C. Halleskog and G. Schulte , “ wnt‐3A and wnt‐5A Counteract Lipopolysaccharide‐Induced Pro‐Inflammatory Changes in Mouse Primary Microglia,” Journal of Neurochemistry 125 (2013): 803–808, 10.1111/jnc.12250. 23534675

[62] T. Clifford , Z. Finkel , B. Rodriguez , A. Joseph , and L. Cai , “Current Advancements in Spinal Cord Injury Research—Glial Scar Formation and Neural Regeneration,” Cells 12 (2023): 853, 10.3390/cells12060853. 36980193 PMC10046908

[63] L. Perez‐Gianmarco and M. Kukley , “Understanding the Role of the Glial Scar Through the Depletion of Glial Cells After Spinal Cord Injury,” Cells 12 (2023): 1842, 10.3390/cells12141842. 37508505 PMC10377788

[64] Y. Hou , J. Luan , T. Huang , et al., “Tauroursodeoxycholic Acid Alleviates Secondary Injury in Spinal Cord Injury Mice by Reducing Oxidative Stress, Apoptosis, and Inflammatory Response,” Journal of Neuroinflammation 18 (2021): 216, 10.1186/s12974-021-02248-2. 34544428 PMC8454169

