# Supplementary material for: Wnt5a‐Induced Exosomes From Bone Marrow Mesenchymal Stem Cells Promote Spinal Cord Injury Repair by Modulating Immune Cell Phenotypes and Alleviating Neuroinflammation via the NF‐κB Pathway
Source: CNS Neurosci Ther. 2026 Mar 26;32(4):e70834. doi: 10.1002/cns.70834 (PMC13140915; doi:10.1002/cns.70834)
Supplement: Supplementary file 1 — Data S1: cns70834‐sup‐0001‐supinfo.doc. [file CNS-32-e70834-s001.doc]

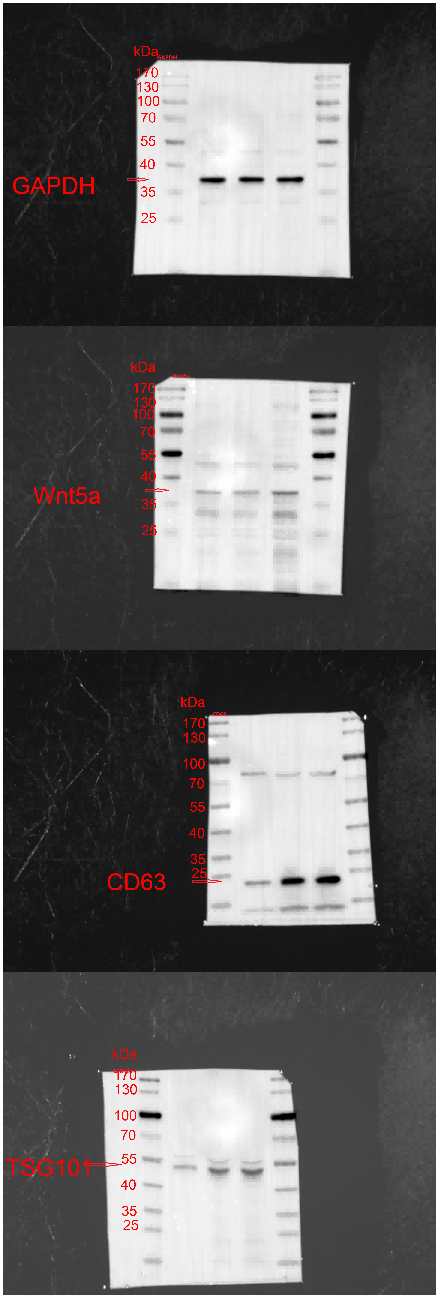


Figure S1. Identification of exosomes using Western blot.

**
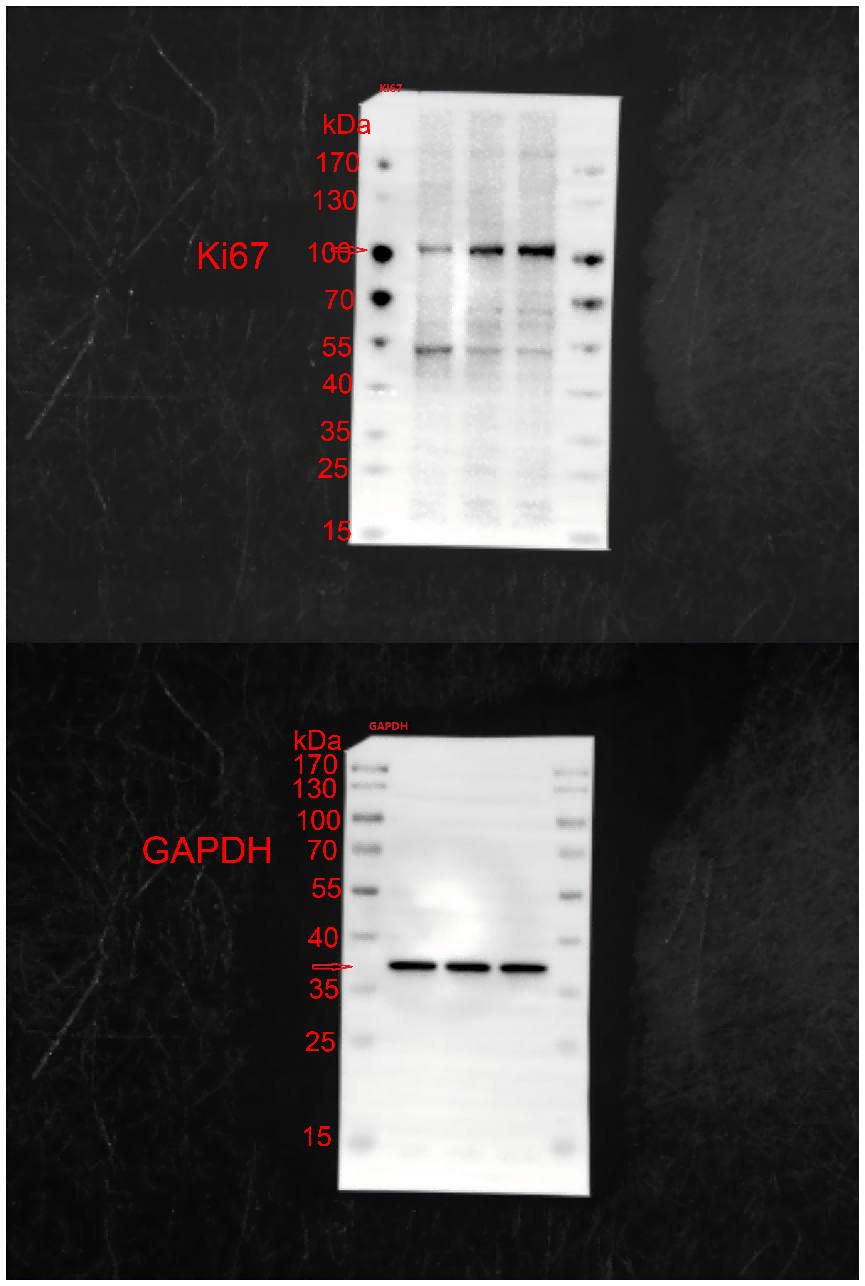
**

Figure S2. The expression of Ki67 protein in the cells was detected by Western blot

**
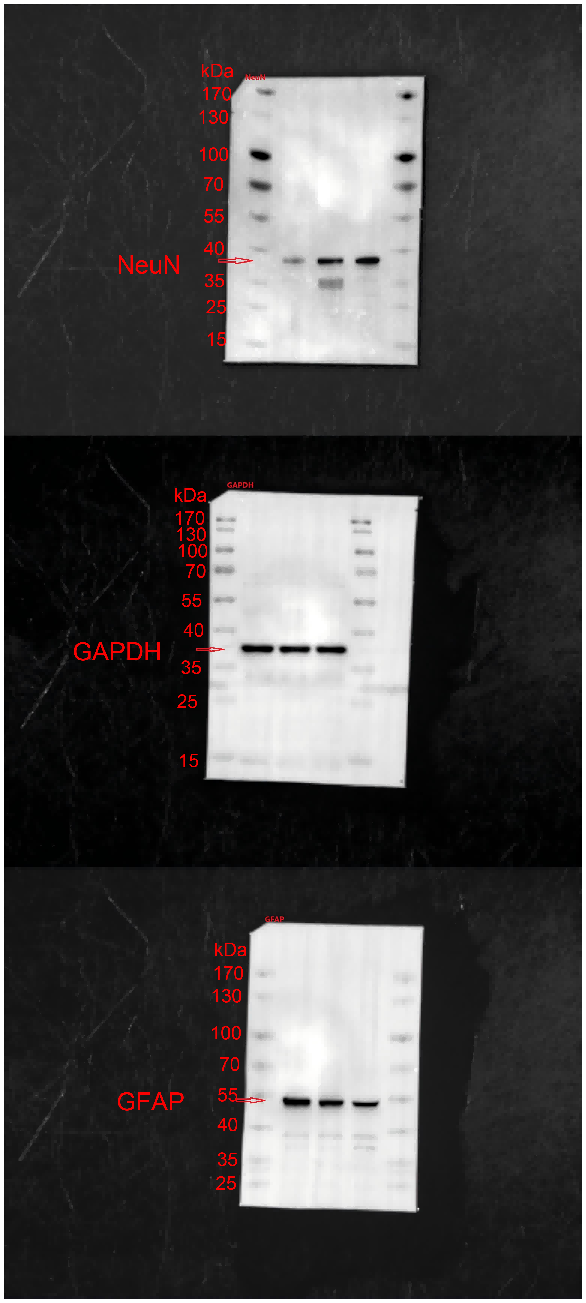
**

Figure S3. The expression of NeuN and GFAP proteins in the cells were examined by Western blot.


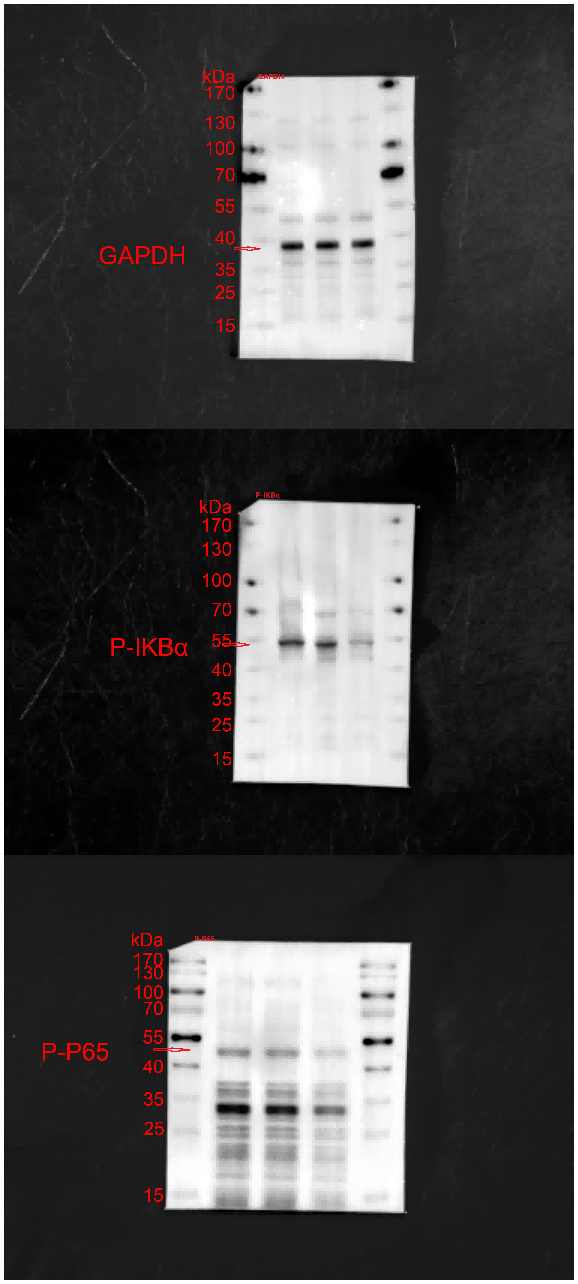


Figure S4. The expression of P65 and IκBα and their phosphorylation in cells from the Con, Exo, and pLV-Exo-Wnt5a groups were detected by Western blot.


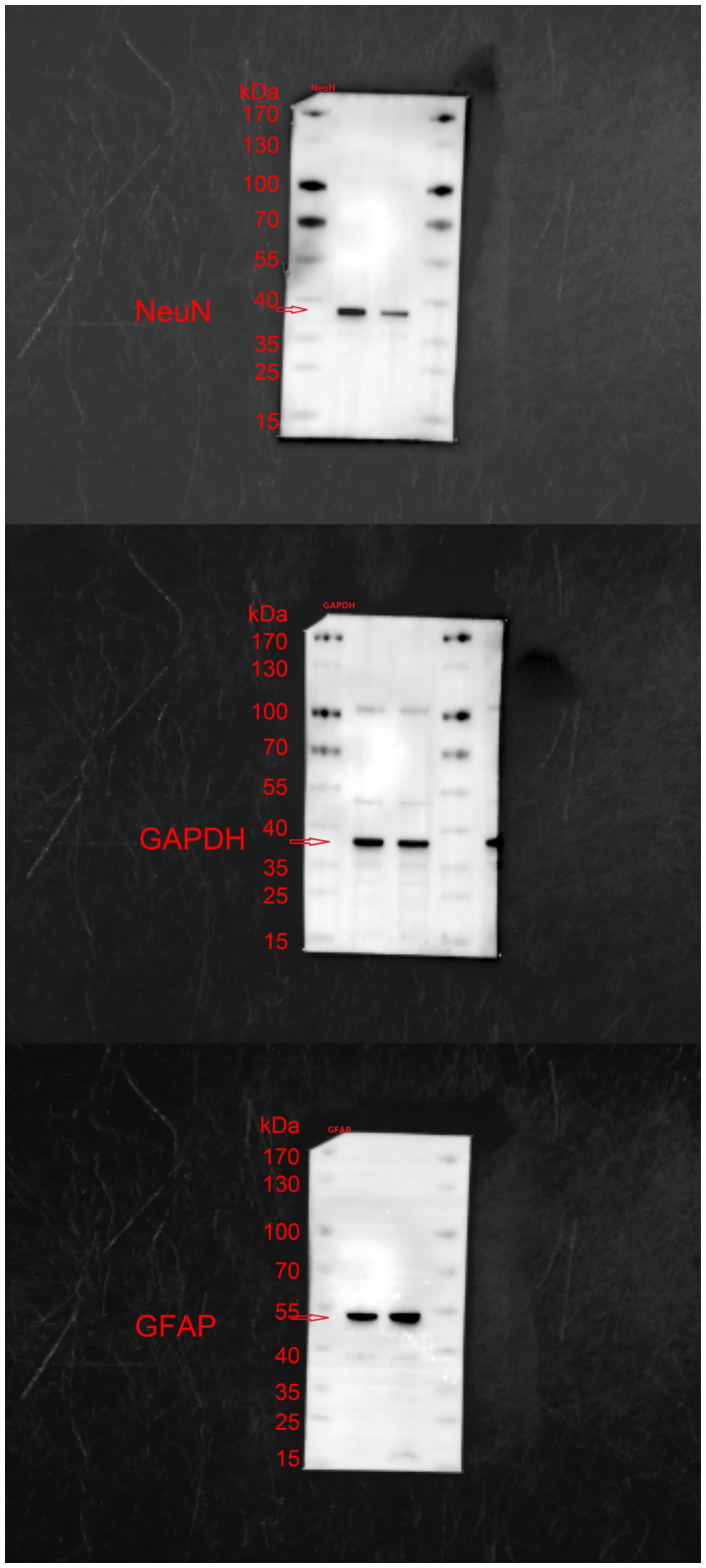


Figure S5. After intervention with the LPS, the expression levels of NeuN and GFAP were determined using Western blot (without LPS as a control).

**
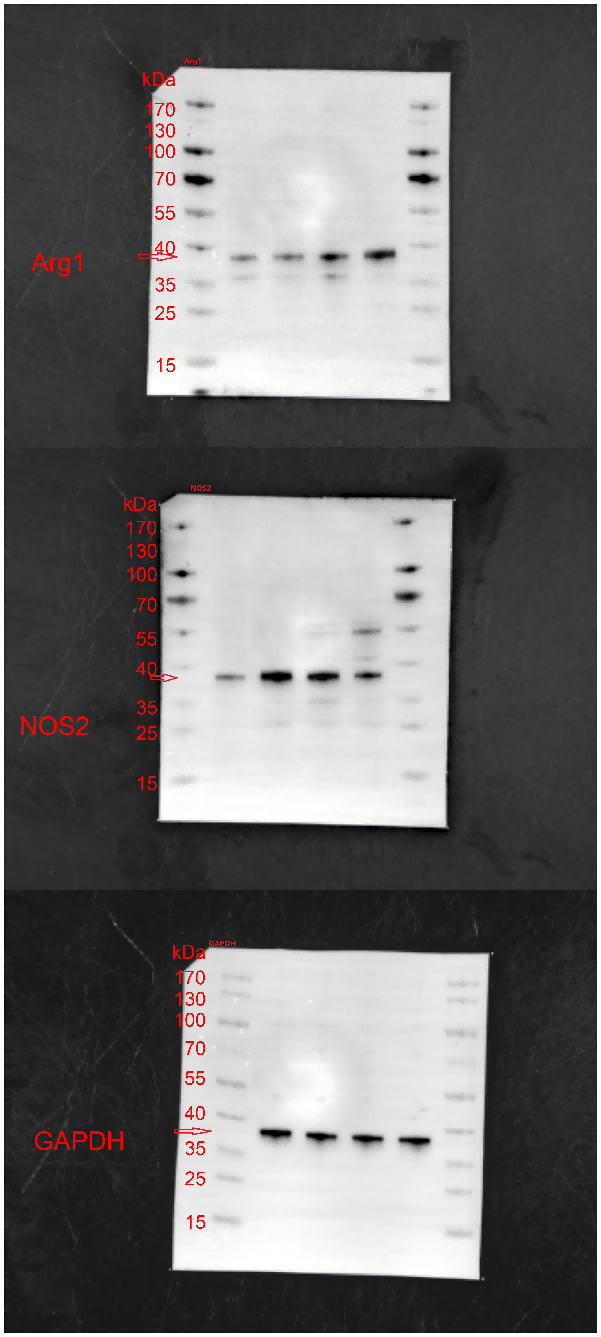
**

Figure S6. Expression levels of Arg1 and NOS2 were measured by Western blot.

**
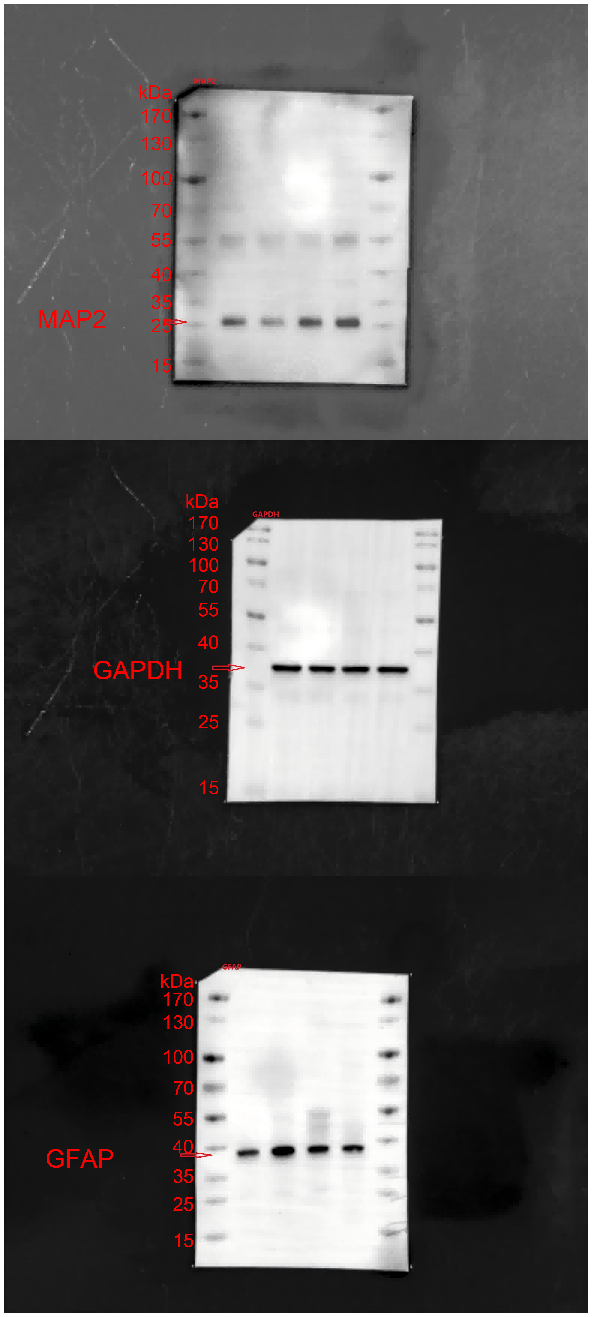
**

Figure S7. The expression levels of MAP2 and GFAP in spinal cord tissues from different groups were determined by Western blot.

**
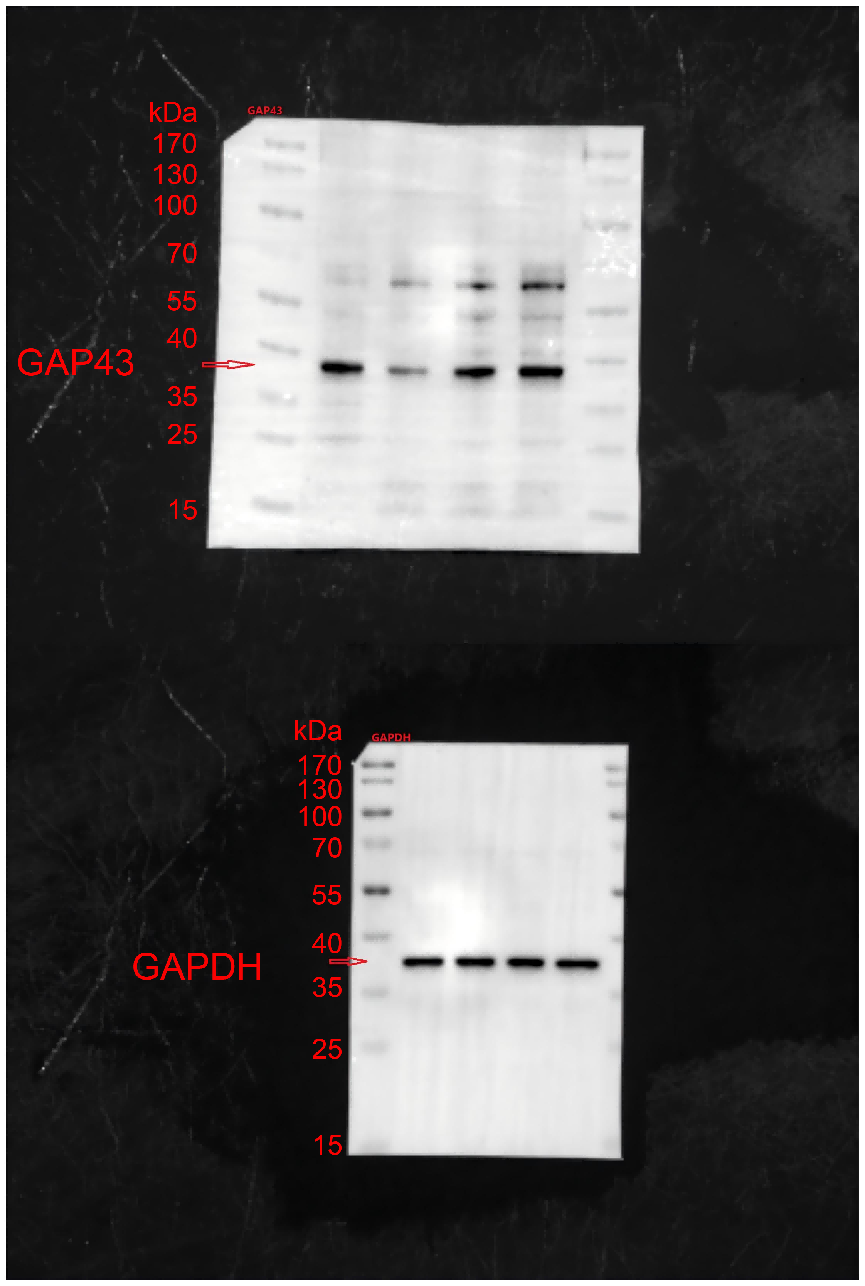
**

Figure S8. The expression levels of GAP43 in spinal cord tissues of different groups were determined by Western blot.
